# Benefits of herbal formulae containing Poria cocos (*Fuling*) for type 2 diabetes mellitus: A systematic review and meta-analysis

**DOI:** 10.1371/journal.pone.0278536

**Published:** 2022-12-01

**Authors:** Yuan Ming Di, Lu Sun, Chuanjian Lu, Xin Feng Guo, Xianyu Tang, Anthony Lin Zhang, Guanjie Fan, Charlie Changli Xue

**Affiliations:** 1 The China–Australia International Research Centre for Chinese Medicine, School of Health and Biomedical Sciences, STEM College, RMIT University, Bundoora, Victoria, Australia; 2 Guangdong Provincial Hospital of Chinese Medicine, Guangdong Provincial Academy of Chinese Medical Sciences, and The Second Clinical College, Guangzhou University of Chinese Medicine, Guangzhou, Guangdong Province, PR China; Institute of medical research and medicinal plant studies, CAMEROON

## Abstract

**Background:**

*Poria cocos* (Schw.) Wolf or *Fuling* is one of the top 10 most frequently prescribed herbs in China for the treatment of type 2 diabetes mellitus (T2DM).

**Objective:**

The purpose of this systematic review is to determine the additional benefit of *Fuling* formulae use in addition to hypoglycaemic agents for T2DM in randomised clinical trials.

**Methods:**

English (5) and Chinese (4) medical databases were searched from their inception to August 2021. RCTs that included *Fuling* in herbal formulae for T2DM were included. Risk of bias were assessed using the Cochrane Collaboration’s procedures. Stata software (13.0) was used for data analysis.

**Results:**

Seventy-three RCTs (6,489 participants) with herbal formulae containing *Fuling* were included. Most studies were at risk of bias and strength of the evidence were low to moderate. Meta-analysis findings showed that the addition of formulae containing *Fuling* to hypoglycaemic agent-treatments could benefit people with T2DM by reducing fasting blood glucose (MD -0.82 [-0.93, -0.71]; I^2^ = 79.6%, *P* = 0.00), 2-hour postprandial blood glucose (MD-1.15 [-1.31, -0.98], I^2^ = 80%, *P* = 0.00) and haemoglobin A1c (MD-0.64 [-0.75, -0.53], I^2^ = 84.7%, *P* = 0.00). Adverse events were also significantly lower in the integrative group than in the hypoglycaemic alone group (RR 0.99 [0.93, 1.06], *P* = 0.87).

**Conclusion:**

Evidence from this study supports the use of *Fuling* formulae combined with hypoglycaemic agents for T2DM. The combined therapies appear to be well tolerated.

**Trail registration:**

This review is registered with the PROSPERO international prospective register of systematic reviews (CRD42020214635).

## 1. Introduction

Diabetes mellitus (DM) is a complex, chronic and progressive condition which is increasing in prevalence at an alarming rate around the world. The International Diabetes Federation reports that 1 in 11 adults have DM, that is about 463 million adults worldwide (1). China is reported to have the largest number of adults with DM (116.4 million or 10.6%) and is predicted to remain so until 2030 [1]. Type 2 diabetes mellitus (T2DM) accounts for approximately 95% of the diabetic population [2].

The risk of T2DM increases in people who have pre-diabetes, where their glucose levels do not meet the criteria for T2DM but are higher than those considered normal [2, 3]. Pre-diabetes is also associated with abdominal obesity, dyslipidaemia and hypertension, which are also risk factors of T2DM. Screening for prediabetes in people who have impaired glucose tolerance is recommended by the American Diabetes Association for the monitoring and development of T2DM [2]. Preventative interventions such as structured lifestyle intervention in people with prediabetes have shown to prevent or delay the development of T2DM [2, 4]. Preventative management can be more beneficial for diabetic people. This coincides with the concept of early prevention of disease in Traditional Chinese Medicine (TCM).

The pathological processes of T2DM is multifaceted, pancreatic beta cell function, obesity, adipose tissue, inflammation all play a role in the development of T2DM. Insulin resistance plays a key role, it is characterised by reduced transport and metabolism of insulin-stimulated glucose in adipocytes and skeletal muscles and impaired suppression of hepatic glucose production [5]. Insulin resistance can also be impacted by fatty acids from engorged visceral adipocytes. Oxidation of fatty acids by muscle and other tissues could inhibit glycolysis and reduce insulin-stimulated glucose removal [6]. Further, inflammation processes also contribute to the pathogenesis and development of T2DM [6, 7]. In adipose tissues, activated macrophages release a variety of molecules that decrease the insulin sensitivity of the adipocytes and further increase their release of proinflammatory cytokines and lead to inflammation [5].

Once T2DM is developed, pharmacological therapies or non-pharmacological interventions are available for treatment. Pharmacological therapies include oral and injectable hypoglycaemic agents. However, common concerns associated with these therapies include hypoglycaemia, gastrointestinal discomfort and inconvenience of injectable insulin. In China, treatment information of TCM for T2DM is presented in the Chinese Diabetes Society Guidelines, with more doctors seeking diverse treatments for diabetes including the use of TCM [8].

In TCM, DM belongs to the category of disease called “*xiao ke*”. Disease characteristics include thirst, excessive drinking, polyuria and weight loss. Poria or *Fuling* (*Poria cocos* (Schw.) Wolf) is an edible and medicinal mushroom and has been widely used in the treatment of *xiao k*e or diabetes in TCM for many years. Among 30 antidiabetic formulas approved by the State Food and Drugs Administrator of China, *Fuling* was one of the top 10 most frequently prescribed herbs [9]. In another study on treating diabetes with TCM, *Fuling* was said to show hypoglycaemic functions via the function of “draining water” [10]. *Fuling* compound groups include triterpenes and polysaccharides [11, 12]. Since *Fuling* is a commonly prescribed herb for T2DM and shows hypoglycaemic functions, we would like to assess its benefits in people with T2DM. However, in TCM, the use of one single herb is uncommon and herbs are often prescribed together as a formula to treat diseases.

The purpose of this systematic review is to determine the additional benefit of *Fuling* formula’s use in addition to hypoglycaemic agents for T2DM in randomised clinical trials.

## 2. Methods

This review complies with the Cochrane systematic review guidelines. The review is registered with the PROSPERO international prospective register of systematic reviews (CRD42020214635).

### 2.1. Search strategy

We searched English- and Chinese-language databases following the methods outlined in the Cochrane Handbook of Systematic Reviews [13]. English-language databases included PubMed, Excerpta Medica Database (Embase), Cumulative Index of Nursing and Allied Health Literature (CINAHL), Cochrane Central Register of Controlled Trials (CENTRAL), including the Cochrane Library, and Allied and Complementary Medicine Database (AMED); and Chinese-language databases included China SinoMed Literature, China National Knowledge Infrastructure (CNKI), Chongqing VIP (CQVIP) and Wanfang. Databases were searched from inception to August 2021. No restrictions were applied.

The literature search was composed of search terms categorised into three blocks: ‘Chinese herbal medicine’, ‘type 2 diabetes mellitus’, and ‘randomised controlled trial’. The search results from each block were then combined using the AND Boolean operator. Search syntax for each database can be found in Supplementary Table 1.

**Table 1:** Characteristic of the included randomised controlled trials.

Study No.	Author Year	No. of patients (I)	No. of patients (C)	Mean age (y) (I)	Mean age (y) (C)	Treatment duration (weeks) Follow-up (weeks)	Treatment	Comparator	Syndrome Differentiation	Outcomes
1	Bai JL 2016 a	100	100	NS	NS	12	SMF+Bigu	Bigu	NS	FBG, 2HPG, HBA1C
2	Bai JL 2016 b	46	46	47	48	12	SMF+Bigu	Bigu	NS	FBG, 2HPG, HBA1C, FINS, IR
3	Chen XP 2021	30	30	56.8	56.1	8	SMF+Bigu	Bigu	qi and yin deficiency	FBG, 2HPG, HBA1C, BMI, TG, TC, LDL, HDL
4	Cui CW 2020	40	40	61.78	61.59	4	Chaihuliujunzi Formula+Basic treatment	Basic treatment	NS	FBG, 2HPG, HBA1C, AE
5	Cui JH 2006	40	40	56.75	52.5	8 (2 weeks)	SMF+Bigu+Sulf	Bigu+Sulf	qi and yin deficiency	FBG, 2HPG, HBA1C
6	Cui YM 2011	35	34	47.11	47.68	8	SMF+Bigu+a-Glucosidase	Bigu+a-Glucosidase	Liver qi stagnation	FBG, 2HPG, HBA1C, TG
7	Ding Y 2019	39	35	53.2	52.7	NS	Jiaweiqiling Formula+Bigu+Insulin	Bigu+Insulin	qi and yin deficiency	FBG, 2HPG, HBA1C, TG, TC, LDL, HDL
8	Du FZ 2020	45	45	64.32	64.48	12	Liuweidihuangwan+insulin	insulin	qi and yin deficiency	FBG, 2HPG, HBA1C, IR
9	Duan G 2015 a	78	78	56.83	55.25	4	Didang Formula+insulin	insulin	phlegm-dampness and blood stasis	FBG, AE
10	Duan G 2015 b	72	72	52.43	54.78	8	Didang Formula+insulin	insulin	phlegm-dampness and blood stasis	FBG, HBA1C, FINS, IR, IS
11	Duan JN 2012	37	37	57.75	56.25	12	Qinxinlianzi Formula+Bigu	Bigu	qi and yin deficiency	FBG, 2HPG, HBA1C, TG, TC, BMI, AE
12	Fan XF 2019	47	47	50.18	51.56	12	Liuweidihuang Pill+Bigu/Sulf	Bigu	NS	FBG, 2HPG, HBA1C, TG, TC, LDL, HDL
13	Fan YL 2014	40	40	NS	NS	12	Shenqijiangtang Granule+Bigu	Bigu	qi and yin deficiency	FBG, 2HPG, HBA1C
14	Fang LL 2015	30	30	NS	NS	8	SMF+Bigu	Bigu	damp heat	FBG, 2HPG, HBA1C, TG, TC, LDL, HDL, AE
15	Fu YH 2016	41	40	NS	NS	8	Gegenqinlianxiaoke Formula+Bigu+Basic treament	Bigu+Basic treament	damp heat	FBG, 2HPG, HBA1C, TG, TC, LDL, HDL, AE
16	Gao L 2017	90	90	58.9	57.6	12	Shenqijiangtang Capsule+Bigu	Bigu	NS	FBG, 2HPG, HBA1C, TG, TC, LDL, HDL
17	Guo FF 2018	42	42	NS	NS	12	Xiaokeshu Pill+Bigu	Bigu	NS	FBG, 2HPG, HBA1C
18	Hou YH 2011	30	30	46.1	46.7	8	SMF+Bigu+a-Glucosidase	Bigu+a-Glucosidase	Spleen deficiency	FBG, 2HPG, HBA1C, TG, TC, LDL, HDL, IR, AE
19	Huang HF 2020	46	46	39.7	39.4	12	Jiangtangning Capsule+a-Glucosidase	a-Glucosidase	qi and yin deficiency	FBG, 2HPG, HBA1C, IR, AE
20	Huang JC 2020	49	49	54.49	51.04	12	Jinlida Granue+insulin	insulin	NS	FBG, 2HPG, HBA1C, AE
21	Huang XT 2013	50	50	NS	NS	4	SMF+Bigu	Bigu	yin deficiency and heat prosperity	FBG, 2HPG
22	Jiang WJ 2012	30	30	NS	NS	8	SMF+Sulf	Sulf	qi and yin deficiency	HBA1C, TG, TC, FINS
23	Jin HY 2019	44	43	50.42	49.82	2	Jianpiziyin xiaoke Formula+Insulin	insulin	qi and yin deficiency	FBG, 2HPG, IS, AE
24	Jin Z 2017	39	39	59.4	59	12 (4 weeks)	Tangshenle Pill+Bigu	Bigu	qi and yin deficiency	FBG, 2HPG, HBA1C, TG, TC, LDL, HDL, AE
25	Li CL 2019	40	40	50.16	50.33	12	Yiqijianpi Formula+Bigu	Bigu	Spleen deficiency	FBG, 2HPG, HBA1C, AE
26	Li JH 2016	30	30	64.18	66.33	4	SMF+Bigu+Sulf	Bigu+Sulf	phlegm-dampness and blood stasis	FBG, 2HPG, TG, TC, LDL, HDL
27	Li S 2020	42	41	41.47	40.85	12	Antangyin+Bigu	Bigu	Spleen deficiency	FBG, 2HPG, TG, TC, LDL, HDL, FINS
28	Li X 2021	54	54	51.4	51.6	12	SMF+Bigu	Bigu	Spleen deficiency	FBG, 2HPG, HBA1C, TG, TC, LDL, HDL, FINS
29	Li XL 2014	30	30	NS	NS	4	SMF+Bigu+a-Glucosidase	Bigu+a-Glucosidase	Spleen deficiency	FBG, 2HPG, HBA1C, AE
30	Li XN 2020	44	44	45.7	46.1	8	Jinlida Granule+Bigu	Bigu	NS	FBG, 2HPG, HBA1C
31	Li ZQ 2011	30	30	51.33	50.46	12	SMF+Meglitinides	Meglitinides	phlegm-dampness and blood stasis	FBG, 2HPG, HBA1C, FINS, IR, AE
32	Lian FM 2015	96	96	NS	NS	12	Jinlida Granule+Bigu	Bigu+placebo	NS	FBG, 2HPG, HBA1C, IR
33	Liu XF 2012	30	30	44.7	45	12	SMF+Meglitinides	Meglitinides	qi and yin deficiency	FBG, 2HPG, HBA1C, TG, TC, LDL, HDL, AE
34	Liu Y 2010	30	30	57.3	56.7	12	SMF+Bigu+Sulf	Bigu+Sulf	qi and yin deficiency	FBG, 2HPG, HBA1C, TG, TC, LDL, HDL, AE
35	Lou XL 2005	52	50	53.5	54	8	SMF+Bigu	Bigu	NS	FBG, 2HPG, HBA1C
36	Lu JZ 2009	30	30	71.35	70.32	4	SMF+Bigu+Sulf	Bigu+Sulf	NS	FBG, 2HPG
37	Lu P 2021	30	30	55.70	54.85	24	Yiqiyangyintiaotangyin+Bigu	Bigu	qi and yin deficiency	FBG, 2HPG, HBA1C, TG, TC, LDL, HDL
38	Luo LD 2009	38	35	51.73	52.16	4	SMF+Bigu+Sulf	Bigu+Sulf	NS	FBG, 2HPG, TG, TC, LDL, HDL, FINS, IR, IS, AE
39	Ma D 2016	33	33	44.7	45.2	12	SMF+TZDs	TZDs	Spleen deficiency	FBG, 2HPG, HBA1C, TG, TC, FINS, IS, AE
40	Mei C 2019	80	80	49.87	50.13	4	Shengmai Formula +Liuweidihuang Formula+TZDs/Bigu	TZDs/Bigu	qi and yin deficiency	FBG, 2HPG, HBA1C, TG, TC, LDL, HDL, FINS, IR, AE
41	Qin Y 2014 a	42	38	NS	NS	12	SMF+Bigu	Bigu	qi and yin deficiency	FBG, 2HPG, FINS, IS, AE
42	Qin Y 2014 b	43	43	49.4	48.8	12	SMF+Meglitinides+insulins	Meglitinides+insulins	yin deficiency and heat prosperity	FBG, 2HPG, HBA1C
43	Shao WY 2020	31	30	55.48	53.77	8	SMF+Bigu	Bigu	qi and yin deficiency	FBG, 2HPG, HBA1C, FINS, IR, AE
44	She WJ 2012	38	34	NS	NS	4	Shuzheng Granule+a-Glucosidase	a-Glucosidase	phlegm-dampness and blood stasis	FBG, HBA1C, TG, TC, LDL, HDL, AE
45	Song YZ 2017	30	30	55.1	56	12	Jinlida Granule+Bigu	Bigu	NS	FBG, 2HPG, HBA1C, BMI, AE
46	Tang Y 2015	69	69	51.6	50.4	12	Liuweidihuang Pill+Bigu	Bigu	yin deficiency and heat prosperity	FBG, 2HPG, HBA1C, TG, TC, LDL, HDL, FINS, IR, AE
47	Wan H 2021	48	49	49.59	49.65	12	Yuquanwan + sodium-glucose co-transporter 2 inhibitor	sodium-glucose co-transporter 2 inhibitor	Spleen deficiency	FBG, 2HPG, HBA1C, FINS, IR, AE
48	Wang DW 2007	56	40	56.5	56.8	8	SMF+Bigu	Bigu	NS	FBG, FINS, IS
49	Wang GM 2009	29	27	59.12	59.16	8	SMF+Bigu+Sulf	Bigu+Sulf	NS	FBG, 2HPG, HBA1C, TG, TC, LDL, HDL, IS
50	Wang JX 2021	45	45	55.24	56.34	2	SMF+Bigu	Bigu	qi and yin deficiency	FBG, 2HPG, HBA1C, QOL, FINS, IR, AE
51	Wang L 2009	30	30	54.17	53.77	12	SMF+Sulf	Sulf	qi and yin deficiency	FBG, 2HPG, HBA1C, TG, TC
52	Wang M 2020	50	50	50.39	50.26	12	SMF+Bigu	Bigu	Spleen deficiency	FBG, 2HPG, HBA1C, AE
53	Wang ML 2012	30	30	68.23	67.26	2	SMF+Insulin	Insulin	qi and yin deficiency	FBG, HBA1C, TG, TC, LDL, HDL, AE
54	Wang SL 2010	30	30	50	53.7	4	Zhibodihuang Formula+Sulf	Sulf	yin deficiency and heat prosperity	FBG, FINS, FINS, IR, IS
55	Wang YX 2010	60	60	51.5	51.3	8	SMF+Bigu+Meglitinides	Bigu+Meglitinides	qi and yin deficiency	FBG, 2HPG, HBA1C
56	Wang ZG 2020	64	62	50.53	50.19	12	Shenqijiangtang Granule+Bigu	Bigu	NS	2HPG, HBA1C, IR
57	Wen LX 2010	58	50	39.2	38.3	4	Liuweidihuang Pill+Bigu+a-Glucosidase	Bigu+a-Glucosidase	yin deficiency and heat prosperity	FBG, 2HPG, TG, TC
58	Xu XJ 2015	20	20	53.5	54.6	12	Shenlinbaizhu Formula+insulin	insulin	Spleen deficiency	BMI
59	Ye WP 2016	50	50	52.13	51.29	12	SMF+insulin	insulin	qi and yin deficiency	FBG, 2HPG, HBA1C, FINS, IR
60	Yu CB 2013	40	40	56.2	55.4	12	Taipingtangke Pill+Bigu	Bigu	qi and yin deficiency	FBG, 2HPG, HBA1C
61	Yu CZ 2017	36	36	NS	NS	12	Jiaweiliujunzi Formula+Bigu+a-Glucosidase	Bigu+a-Glucosidase	Spleen deficiency	FBG, 2HPG, HBA1C, FINS, IR, HCP
62	Yu Y 2020	48	48	53.48	53.21	12	SMF+Bigu	Bigu	damp heat	FBG, 2HPG, HBA1C
63	Yuan HL 2019	53	53	65.1	65.3	6	Yiqiyangyinqingrehuayu Formula+Bigu	Bigu	qi and yin deficiency	FBG, 2HPG, HBA1C
64	Zhang AX 2012	46	46	NS	NS	6	Yunvjian Formula+a-Glucosidase	a-Glucosidase	Spleen deficiency	FBG, 2HPG, HBA1C, TG, TC, HDL
65	Zhang GZ 2002	100	50	52.6	50.5	8	SMF+Bigu+Sulf	Bigu+Sulf	NS	FBG, 2HPG, TG, TC, LDL, HDL, FINS, HCP, AE
66	Zhang L 2019	60	60	57.13	57.46	12	Shenqijiangtang Granule+Bigu+Sulf	Bigu+Sulf	qi and yin deficiency	FBG, 2HPG, HBA1C, TG, TC, LDL, HDL
67	Zhang N 2013	50	45	52.33	54.23	12	Jiaweishenqidihuang Formula+Bigu	Bigu	qi and yin deficiency	FBG, HBA1C, TG, TC, LDL, HDL, FINS, IR, BMI
68	Zhang XJ 2012	30	30	83.65	85.82	25	SMF+Meglitinides+insulins	Meglitinides+insulins	yin deficiency and heat prosperity	FBG, 2HPG, HBA1C
69	Zhou JJ 2012	30	30	59.23	58.63	4	Yiqiyangyin Capsule+Sulf	Sulf	qi and yin deficiency	FBG, 2HPG, HBA1C
70	Zhu L 2020	30	30	49.35	48.75	24	Qishan Granule+Bigu	Bigu	damp heat	FBG, 2HPG, HBA1C, LDL
71	Zhu LY 2015	35	35	51.73	52.95	12	SMF+Bigu+Sulf	Bigu+Sulf	yin deficiency and heat prosperity	FBG, 2HPG, AE
72	Zhu ZZ 2009	49	49	52.5	53.1	8	SMF+Bigu+Sulf	Bigu+Sulf	NS	FBG, FINS
73	Zhuo J 2015	37	38	57.41	58.11	12	Bushenkangshuai Tablet+TZDs+a-Glucosidase	TZDs+a-Glucosidase	phlegm-dampness and blood stasis	FBG, 2HPG, HBA1C

Abbreviations: 2HPG, 2 hour postprandial blood glucose; AE, adverse events; Bigu, Biguanides; BMI, body mass index; FBG, fasting blood glucose; FINS, fasting insulin; HBA1C, glycated haemoglobin; IR, Homeostatic model assessment of insulin resistance; IS, insulin resistance index; LDL, low-density lipoprotein; HDL, high-density lipoprotein; SMF, self made formula; Sulf, Sulfonylureas; TC, total cholesterol; TG, triglyceride.

We also searched reference lists of previous systematic reviews and included studies. Clinical trial registries were also searched including the Australian New Zealand Clinical Trial Registry (ANZCTR), Chinese Clinical Trial Registry (ChiCTR), European Union Clinical Trials Register (EU-CTR) and USA National Institutes of Health register (ClinicalTrials.gov). When required, we contacted trial investigators by email or telephone to obtain data. If we didn’t receive a response after four weeks, we marked the unknown information ‘not available’.

### 2.2. Study inclusion criteria

#### 2.2.1 Study design

Randomised controlled trials (RCTs) were eligible.

#### 2.2.2 Participants

Adults diagnosed with T2DM using the following guidelines: 1999 World Health Organization (WHO) [14], Chinese Diabetes Society [15, 16], American Diabetes Association [17], or a description of diagnostic criteria including:

- Fasting blood glucose (FBG, defined as no caloric intake for at least 8 hours) ≥126 mg/dL (7.0 mmol/L),
- Or 2-h plasma glucose (PG) ≥200mg/dL (11.1 mmol/L) during an oral glucose tolerance test (OGTT). The test should be performed as described by the WHO, using a glucose load containing the equivalent of 75 g anhydrous glucose dissolved in water.
- Or HbA1c ≥6.5% (48mmol/mol). The test should be performed in a laboratory using a method that is National Glycohemoglobin Standardization Program certified and standardized to the Diabetes Control and Complications Trial assay.
- Or in a patient with classic symptoms of hyperglycemia or hyperglycemic crisis, a random plasma glucose ≥200 mg/dL (11.1 mmol/L). In the absence of unequivocal hyperglycemia, results should be confirmed by repeat testing.

#### 2.2.3 Type of interventions

Chinese herbal medicine (CHM) and integrative medicine such as CHM plus hypoglycaemic drugs; CHM interventions should include *Fuling* as one of the herbs.

#### 2.2.4 Type of controls

Therapies recommended in guidelines, including pharmacotherapy, diet therapy and lifestyle interventions.

#### 2.2.5 Outcomes

The primary outcome measures were:

- Blood glucose tests (fasting blood glucose, post-prandial blood glucose, hemoglobin Hba_1c_);
- Adverse events (AEs);

The secondary outcomes were:

- Blood lipid metabolism indicators (triglyceride, cholesterol, low-density lipoprotein, high-density lipoprotein);
- β-cell function indicators: fasting serum insulin (Fins) the unit of FINS is pmol/L or μu/ml will be included (pmol/L = μu/ml ×6.965); HOMA-IR(IR) = FBG×FINS/22.5; HOMA-IS (IS) = 1/(Fins x FPG);
- Body Mass Index (BMI).

### 2.3. Study exclusion criteria

- Quasi-randomized controlled trials;
- Prediabetic state;
- Type 1 diabetes;
- Gestational diabetes;
- Other specific types of diabetes included in the American Diabetes Association and Chinese Diabetes Society:
- Genetic defects of beta-cells
- Genetic defects in insulin action
- Diseases of the exocrine pancreas
- Endocrinopathies
- Drug or chemical induced diabetes
- Infections
- Uncommon forms of immune-mediated diabetes
- Other genetic syndromes sometimes associated with diabetes
- Diabetic complications and comorbidities;
- Integrative medicine studies that used different therapies in the intervention group and control group;
- If the control group uses a form of Chinese medicine.

### 2.4. Data screening, extraction and management

Search results were synthesized by removing duplicates, followed by screening of titles and abstracts by LS and YMD. Full texts were obtained and screened by two reviewers (LS and YMD). Eligible studies satisfying the inclusion criteria were extracted using EpiData software (EpiData Association, Odense, Denmark). RCTs that used a CHM formula which included *Fuling* as one of the herbs were included. LS and YMD extracted the data from included studies independently and double-checked the data to obtain information on authors, publication year, title, journal, participants’ characteristics, sample size, methodological details, intervention details, treatment duration, outcome measures and AEs. Study characteristics are tabulated and presented.

### 2.5. Assessment of risk of bias in included studies

Risk of bias was assessed using the Cochrane Collaboration’s procedures [13]. RevMan software (Version 5.2.4, Copenhagen: The Nordic Cochrane Centre, The Cochrane Collaboration, 2012) was used for risk of bias analysis. Items of bias assessed included sequence generation, allocation concealment, blinding of participants, blinding of personnel, blinding of outcome assessment, incomplete outcome data, selective outcome reporting and other bias such as baseline imbalance and funding. Risk of bias assessment was conducted by two independent reviewers (LS and YMD) and disagreement was resolved by discussion or consultation with a third person (ALZ).

### 2.6. Data analysis

Continuous outcomes were presented as mean difference (MD) with 95% confidence interval (CI) between two groups, whereas dichotomous data were presented as relative risk (RR) with 95% CI. Stata software (13.0) was used for data analysis. Considering heterogeneities among trials, all meta-analysis was performed with a random effects model. Heterogeneities between studies was estimated by *I*^2^. An I^2^ score greater than 50% was considered to indicate substantial heterogeneity.

Subgroup analysis were performed where possible, including studies with low risk for sequence generation, FBG level at baseline (low 6-≤8 mmol/L, medium 8–10 mmol/L, high ≥10 mmol/L), patient age groups (18–40 years, 41–64 years, >65 years), BMI (normal <24kg/m^2^, overweight ≥24-28kg/m^2^, obese≥28kg/m^2^), disease duration (<5 years, ≥5-10years, ≥10 years), treatment duration (short ≤3 months, medium 3–6 months and long ≥ 6 months), comparator drugs class and TCM syndrome differentiation (one of the fundamental principle of TCM to guide diagnosis and treatment).

Where possible, meta-regression were used to explore the source of heterogeneity. Sensitivity analysis using the leave-one-out method were also performed by dropping outlier study(ies) in the meta-analysis.

Exploration of publication bias was planned if more than ten studies were included in a meta-analysis. Egger’s test and funnel plots were used to assess publication bias.

The Grading of Recommendations Assessment, Development and Evaluation (GRADE) approach was used to assess the quality of evidence.

## 3. Results

### 3.1. Description of included studies

**Fig 1:**
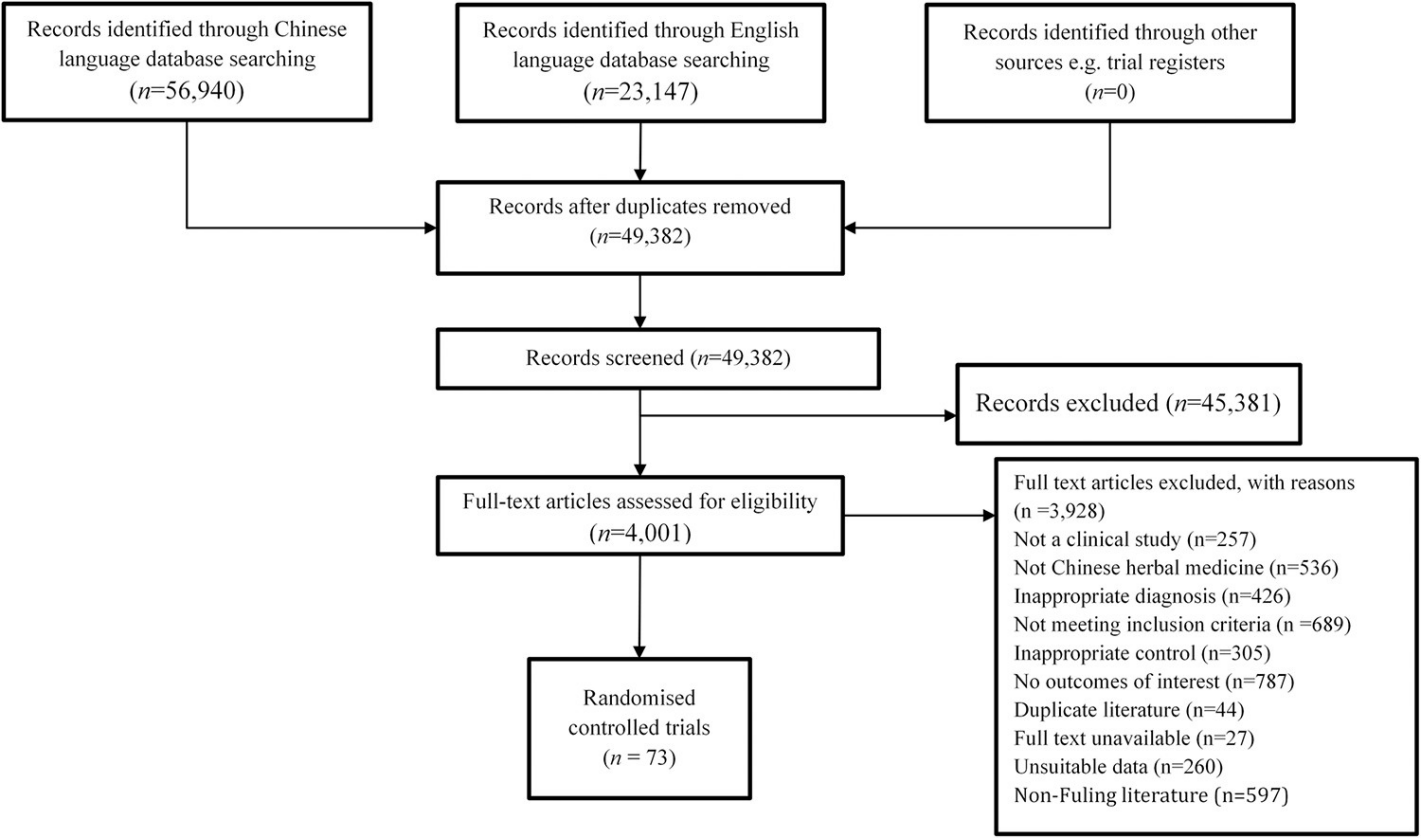
Flow chart of study selection process.

Our search identified 49,382 articles in the included databases, 73 RCTs were included in the systematic review [18–90]. The screening process is shown in Fig 1.

All studies were randomized, parallel-group, controlled trials conducted in China between 2002 and 2021 (Table 1). One study published was in English [54] and the rest in Chinese language. All studies included participants diagnosed in accordance with the WHO, Chinese Diabetes Society or American Diabetes Association diagnostic criteria or clear description of diagnostic criteria for T2DM. In total, 6,489 participants were included in these RCTs, with ages ranging from 20 to 85.82 years. Duration of T2DM ranged from 2 weeks to 20.1 years. Treatment duration ranged from 4 to 25 weeks. Four studies had follow-up periods ranging from 2 weeks to 3 months [20, 36, 52, 87].

This systematic review looked at CHM formulae that contained *Fuling* and other herbs. All included RCTs assessed CHM as integrative medicine for T2DM.

One hundred and thirty-eight herbs were used in the formulas. Besides *Fuling*, the most commonly used herbs in the included clinical trials were *huangqi* (Astragali Radix), *shanyao* (Dioscoreae rhizome), *shengdihuang* (Rehmannaie Radix), *maimendong* (Ophiopogonis Radix), *baizhu* (Atractylodis Macrocephalae Rhizoma), *danshen* (Salviae Miltiorrhizae Radix et Rhizoma), *tianhuafen* (Trichosanthis Radix), *shanzhuyu* (Corni Fructus), *zexie* (Alismatis Rhizoma) and *gegen* (Puerariae Radix).

All studies used the same hypoglycaemic agent in both treatment groups. Different classes of hypoglycaemic agents were used across studies including biguanides, sulfonylureas, α-Glucosidase inhibitors, meglitinides, and insulins. Combinations of hypoglycaemic agents were also used.

TCM syndrome differentiation was described in fifty-six studies. The most common syndromes described in the studies include *qi* and *yin* deficiency, *yin* deficiency and excessive heat, Spleen deficiency, and phlegm-dampness and blood stasis.

### 3.2. Risk of bias in the included studies

The results of the risk of bias judgements for individual studies are presented in Table 2, figures for the overall risk of bias graph are provided in S1 Fig in S1 File and for individual studies are provided in S2 Fig in S1 File. All included studies were described as randomized, 30 trials described the correct process of random sequence generation and was rated low risk of bias for this domain [22, 25, 26, 29–31, 35, 39, 41, 45, 47–49, 52–54, 59, 60, 69, 71, 76, 80–85, 87, 89]. Five trials [19, 23, 27, 46, 55] were assessed as high risk of bias in the domain of sequence generation as they used sequence of visit for randomisation. Three studies provided details on correct allocation concealment method and was rated low risk of bias for allocation concealment [29, 54, 76]. The remaining studies did not provide information on allocation concealment and was judged as unclear risk of bias for the domain of allocation concealment. One trial [54] used placebo in the control group and was judged to have low risk of bias for blinding of participants and personnel. One trial [76] described the trial as single-blind but with no further details so was judged as unclear risk of bias for this domain. The remaining studies were judged as high risk of bias for blinding of participants and personnel as the treatment given in the two groups were obviously different and no blinding was attempted One trial [54] used independent statistician and was judged as low risk of bias for blinding of outcome assessors, the remaining studies were assessed as unclear risk of bias in blinding of outcome assessors due to insufficient information. Included RCTs either had no dropouts or presented information on dropouts are assessed as low risk of bias for the domain of incomplete data. Risk of selective reporting was high in eight studies, they did not report on all outcome measures described in the methods section [18, 20, 25, 50, 52, 60, 66, 76]. In the included RCTs, only one study [54] registered the trial protocol and reported on all planned outcomes and was judged as low risk of bias for selective reporting. The remaining studies were assessed as unclear as there were no trial protocols published or trial registration identified. There were low risk of bias for baseline imbalance and funding for included trials.

**Table 2:** Risk of bias assessments of the included studies.

Study ID	Author Year	Sequence generation	Allocation concealment	Blinding of participants and personnel	Blinding of outcome assessors	Incomplete outcome data	Selective outcome reporting	Other bias (baseline imbalance, funding)
1	Bai JL 2016 a	H	U	H	U	L	U	L
2	Bai JL 2016 b	U	U	H	U	L	H	L
3	Chen XP 2021	U	U	H	U	L	U	L
4	Cui CW 2020	L	U	H	U	L	U	L
5	Cui JH 2006	U	U	H	U	L	H	L
6	Cui YM 2011	U	U	H	U	L	U	L
7	Ding Y 2019	L	U	H	U	L	U	L
8	Du FZ 2020	L	U	H	U	L	U	L
9	Duan G 2015 a	U	U	H	U	L	U	L
10	Duan G 2015 b	H	U	H	U	L	U	L
11	Duan JN 2012	L	U	H	U	L	H	L
12	Fan XF 2019	L	U	H	U	L	U	L
13	Fan YL 2014	H	U	H	U	L	U	L
14	Fang LL 2015	U	U	H	U	L	U	L
15	Fu YH 2016	L	L	H	U	L	U	L
16	Gao L 2017	L	U	H	U	L	U	L
17	Guo FF 2018	L	U	H	U	L	U	L
18	Hou YH 2011	U	U	H	U	L	U	L
19	Huang HF 2020	U	U	H	U	L	U	L
20	Huang JC 2020	L	U	H	U	L	U	L
21	Huang XT 2013	U	U	H	U	L	U	L
22	Jiang WJ 2012	U	U	H	U	L	U	L
23	Jin HY 2019	L	U	H	U	L	U	L
24	Jin Z 2017	U	U	H	U	L	U	L
25	Li CL 2019	U	U	H	U	L	U	L
26	Li JH 2016	U	U	H	U	L	U	L
27	Li S 2020	L	U	H	U	L	U	L
28	Li X 2021	U	U	H	U	L	U	L
29	Li XL 2014	L	U	H	U	L	U	L
30	Li XN 2020	U	U	H	U	L	U	L
31	Li ZQ 2011	L	U	H	U	L	U	L
32	Lian FM 2015	L	L	L	U	L	U	L
33	Liu XF 2012	U	U	H	U	L	U	L
34	Liu Y 2010	U	U	H	U	L	U	L
35	Luo XL 2005	U	U	H	U	L	U	L
36	Lu JZ 2009	L	U	H	U	L	U	L
37	Lu P 2021	L	U	H	U	L	U	L
38	Luo LD 2009	H	U	H	U	L	U	L
39	Ma D 2016	L	U	H	U	L	U	L
40	Mei C 2019	L	U	H	U	L	U	L
41	Qin Y 2014 a	U	U	H	U	L	H	L
42	Qin Y 2014 b	L	U	H	U	L	U	L
43	Shao WY 2020	L	U	H	U	L	U	L
44	She WJ 2012	U	U	H	U	L	U	L
45	Song YZ 2017	L	U	H	U	L	H	L
46	Tang Y 2015	L	U	H	U	L	U	L
47	Wan H 2021	U	U	H	U	L	U	L
48	Wang DW 2007	H	U	H	U	L	U	L
49	Wang GM 2009	U	U	H	U	L	U	L
50	Wang JX 2021	L	U	H	U	L	U	L
51	Wang L 2009	U	U	H	U	L	U	L
52	Wang M 2020	L	U	H	U	L	U	L
53	Wang ML 2012	L	L	U	U	L	H	L
54	Wang SL 2010	U	U	H	U	L	U	L
55	Wang YX 2010	U	U	H	U	L	U	L
56	Wang ZG 2020	U	U	H	U	L	U	L
57	Wen LX 2010	L	U	H	U	L	U	L
58	Xu XJ 2015	L	U	H	U	L	H	L
59	Ye WP 2016	U	U	H	U	L	U	L
60	Yu CB 2013	U	U	H	U	L	U	L
61	Yu CZ 2017	U	U	H	U	L	U	L
62	YU Y 2020	U	U	H	U	L	U	L
63	Yuan HL 2019	U	U	H	U	L	U	L
64	Zhang AX 2012	U	U	H	U	L	U	L
65	Zhang GZ 2002	U	U	H	U	L	H	L
66	Zhang L 2019	U	U	H	U	L	U	L
67	Zhang N 2013	U	U	H	U	L	U	L
68	Zhang XJ 2012	L	U	H	U	L	U	L
69	Zhou JJ 2012	U	U	H	U	L	U	L
70	Zhu L 2020	L	U	H	U	L	U	L
71	Zhu LY 2015	L	U	H	U	L	U	L
72	Zhu ZZ 2009	U	U	H	U	L	H	L
73	Zhuo J 2015	U	U	H	U	L	U	L

Risk of Bias Judgements: L: low risk, U: Unclear risk or no information specified, H: High risk

### 3.3. Effects of intervention

#### 3.3.1. Blood glucose tests

*3*.*3*.*1*.*1*. *Fasting blood glucose (FBG)*. Seventy-one RCTs including 6,389 participants assessed the effects of CHM plus hypoglycaemic agents versus hypoglycaemic agents alone, these studies used the same hypoglycaemic agents in both groups [18–33, 35–59, 61–90]. Treatment duration ranged from 2 to 24 weeks.

**Fig 2:**
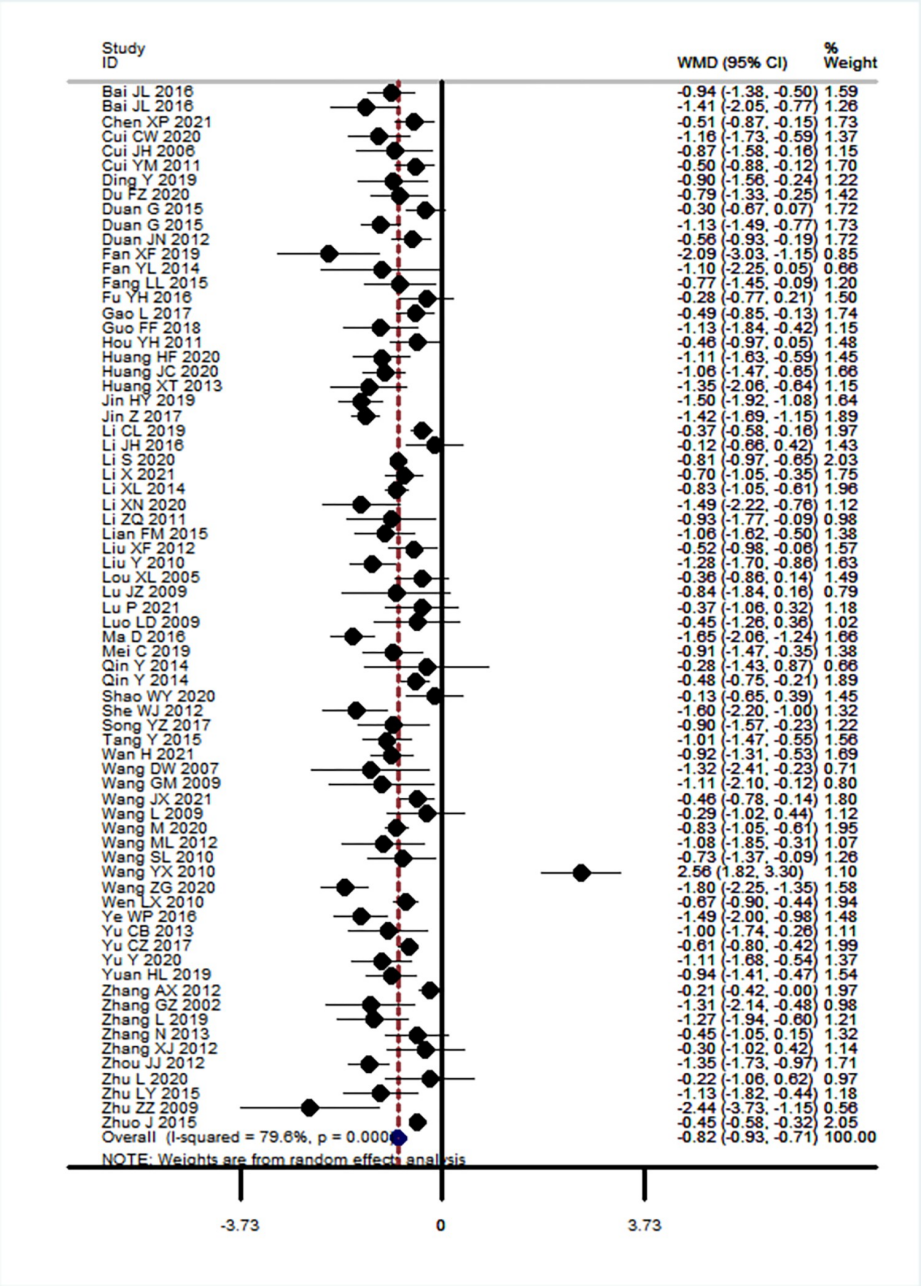
Forest plot of fasting blood glucose level at end of treatment, *Fuling* formulae plus hypoglycaemic agents versus hypoglycaemic agents alone, results of meta-analysis.

The integrative use of CHM plus hypoglycaemic agents was superior to hypoglycaemic agents alone at reducing FBG levels at the end of treatment (MD -0.82 [-0.93, -0.71]; I^2^ = 79.6% *P* = 0.00, Fig 2), high heterogeneity was observed. Meta-regression results showed that baseline FBG levels (*t* = 2.42, *P =* 0.02) and the reporting of TCM syndrome (*t* = 2.51, *P =* 0.02) may be the source of heterogeneity between studies. Sensitivity analysis by removing one outlier study [57] produced results similar to the overall meta-analysis (MD -0.85 [-0.95, -0.75]; I^2^ = 74.1% *P* = 0.00). Egger’s test demonstrated there was evidence of publication bias (*t* = 2.21, *P =* 0.03), funnel plot also showed asymmetry (S3 Fig in S1 File).

Meta-analysis of studies assessed as low risk of bias for sequence generation produced a similar result to the overall results with reduced heterogeneity (29 RCTs, 2,576 participants, MD -0.82 [-0.95, -0.69]; I^2^ = 61.8%, *P* = 0.00).

Subgroup analyses based on comparator drug class, patient age groups, baseline levels of FBG, TCM syndrome differentiation, treatment duration, disease duration and baseline levels of BMI all showed significant differences between groups (Table 3).

**Table 3:** Summary of meta-analysis results and sub-group analysis.

Outcomes (unit)	Group	Subgroup	No. of Studies	MD [95% CI]	I^2^
FBG (mmol/L)	All studies	All studies	71 (6,389)	-0.82 [-0.93, -0.71]*	79.6%
	Risk of bias SG	Low risk of bias SG	29 (2,576)	-0.82 [-0.95, -0.69]*	61.8%
	Comparator drug class	Biguanides	30 (2,811)	-0.85 [-1.00, -0.70]*	71.3%
		Sulfonylureas	3 (180)	-0.85 [-1.49, -0.21]*	73.4%
		Insulin	6 (675)	-1.04 [-1.39, -0.70]*	75%
		Biguanides+ Sulfonylureas	10 (649)	-1.01 [-1.35, -0.66]*	55.3%
		Biguanides+ a-Glucosidase	5 (300)	-0.67 [-0.78, -0.55]*	0.0%
		Biguanides + insulin	1 (74)	-0.90 [-1.56, -0.24]*	NA
	Age of patients	<40 years	2 (200)	-0.83 [-1.24, -0.41]*	56.1%
		41–64 years	46 (4,047)	-0.79 [-0.93, -0.64]*	81.9%
		>65 years	4 (240)	-0.51 [-0.97, -0.05]*	37.2%
	FBG level at baseline	8-10mmol/L	43 (3,942)	-0.89 [-1.03, -0.76]*	76.9%
		≥10mmol/L	20 (1,893)	-0.66 [-0.98, -0.34] *	84.3%
	TCM syndrome differentiation	Qi and yin deficiency	25 (2,087)	-0.75 [-1.01, -0.49]*	86.3%
		Phlegm-dampness and blood stasis	6 (567)	-0.72 [-1.10, -0.34]*	82.6%
		Yin deficiency and excessive heat	7 (622)	-0.74 [-0.96, -0.52]*	41.2%
		Spleen deficiency	10 (818)	-0.72 [-0.92, -0.52]*	84.6%
	Treatment duration	≤6months	68 (6,195)	-0.83 [-0.95, -0.71]*	80.3%
		≥6months	2 (120)	-0.27 [-0.81, 0.28]	0.0%
	Disease duration	<5years	16 (1,290)	-0.70 [-0.98, -0.41]*	87.8%
		≥5-10years	20 (1,716)	-0.88 [-1.10, -0.66]*	82%
		≥10years	5 (420)	-0.65 [-0.87, -0.44]*	31.3%
	BMI level at baseline	≥24-28kg/m^2^	6 (428)	-0.54 [-0.72, -0.36]*	0.0%
2hPG (mmol/L)	All studies	All studies	62 (5,518)	-1.14 [-1.31, -0.98]*	80%
	Risk of bias SG	Low risk of bias SG	26 (2,366)	-1.11 [-1.34, -0.87]*	74.3%
	Comparator Drug class	Biguanides	27 (2,662)	-1.29 [-1.52, -1.06]*	51.5%
		Sulfonylureas	3 (180)	-0.90 [1.57, -0.22]*	18.8%
		Insulin	4 (375)	-1.46 [-2.14, -0.78]*	85%
		Biguanides+ Sulfonylureas	9 (649)	-1.45 [-2.20, -0.70]*	89.4%
		Biguanides+ a-Glucosidase	5 (300)	-0.76 [-0.93, -0.60]*	0.0%
		Biguanides+ insulin	1 (74)	-0.70 [-1.32, -0.09]*	NA
	Age of patients	≤40 years	2 (200)	-1.04 [-1.75, -0.33]*	70%
		41–64 years	47 (4,117)	-1.11 [-1.30, -0.91]*	80.3%
		>65 years	3 (180)	-1.87 [-3.25, -0.49]*	88.0%
	FBG level at baseline	8–10 mmol/L	41 (3,727)	-1.18 [-1.36, -1.00]*	76%
		≥10 mmol/L	14 (1,237)	-1.01 [-1.52, -0.50]*	82.4%
	TCM syndrome differentiation	qi and yin deficiency	22 (1,842)	-0.96 [-1.24, -0.69]*	76.7%
		Yin deficiency and excessive heat	7 (622)	-1.15 [-1.9, -0.60]*	74%
		Spleen deficiency	10 (818)	-1.13 [-1.49, -0.78]*	85.3%
		Phlegm-dampness and blood stasis	3 (195)	-1.46 [-3.14, 0.22]	94.8%
	Treatment duration	≤3months	60 (5,384)	-1.17 [-1.33, -1.00]*	80.6%
		≥6months	1 (60)	-0.50 [-1.35, 0.35]	NA
	Disease duration	<5 years	19 (1,559)	-1.13 [-1.42, -0.84]*	72%
		≥5–10 years	23 (2,004)	-1.16 [-1.46, -0.85]*	86.9%
		≥10 years	4 (360)	-1.12 [-1.64, -0.60]*	82.1%
	BMI level at baseline	≥24–28 kg/m^2^	5 (323)	-0.91 [-1.17, -0.65]*	0.0%
HbA_1c_ (%)	All studies	All studies	58 (5,168)	-0.64 [-0.75, -0.53]*	84.7%
	Risk of bias SG	Low risk of bias SG	24 (2,168)	-0.54 [-0.70, -0.39]*	78.7%
	Comparator Drug class	Biguanides	27 (2,644)	-0.73 [-0.90, -0.56]*	65.4%
		Sulfonylureas	3 (120)	-0.55[-0.91, -0.20]*	0.0%
		Insulin	4 (492)	-0.64 [-0.93, -0.36]*	76.8%
		Biguanides+ Sulfonylureas	4 (316)	-0.70 [-1.31, -0.10]*	80.0%
		Biguanides+ a-Glucosidase	4 (261)	-0.29 [-0.49, -0.09]*	77.5%
		Biguanides+ insulin	1 (74)	-0.90 [-1.50, -0.30]*	NA
	Age of patients	41–64 years	44 (3,881)	-0.68 [-0.81, -0.55]*	84.7%
		>65 years	3 (226)	-0.42 [-1.08, 0.24]	74.6%
	FBG level at baseline	8–10 mmol/L	40 (3,625)	-0.65 [-0.78, -0.52]*	83.0%
		≥10 mmol/L	11 (1,072)	-0.69 [-0.95, -0.42]*	80.3%
	TCM syndrome differentiation	qi and yin deficiency	24 (1,980)	-0.66 [-0.83, -0.48]*	67.9%
		Phlegm-dampness and blood stasis	4 (351)	-0.38 [-0.61, -0.15]*	74.9%
		Spleen deficiency	9 (735)	-0.50 [-0.75, -0.26]*	87.6%
		yin deficiency and excessive heat	3 (284)	-0.62 [-1.21, -0.03]	89.0%
	Treatment duration	≤3months	55 (4,974)	-0.67 [-0.77,-0.56]*	83.8%
		≥6months	2 (120)	-0.04 [-0.14, 0.22]	0.0%
	Disease duration	<5 years	16 (1,329)	-0.64 [-0.85, -0.43] *	84.3%
		≥5–10 years	21 (1,874)	-0.80 [-1.01, -0.58] *	88.6%
		≥10 years	5 (420)	-0.46 [-0.66, -0.26] *	49.3%
	BMI level at baseline	≥24-28kg/m^2^	6 (418)	-0.39 [-0.57, -0.22]*	32.8%
TG (mmol/L)	All studies	All studies	30 (2,571)	-0.34 [-0.40, -0.29]*	69.7%
	Risk of bias SG	Low risk of bias SG	12 (1,178)	-0.30 [-0.39, -0.21]*	75.8%
	Comparator Drug class	Biguanides	11 (1,030)	-0.35 [-0.42, -0.21]*	70.3%
		Sulfonylureas	2 (120)	-0.61 [-0.80, -0.42]*	0.0%
		Insulin	1 (60)	-0.53[-0.79, -0.27]*	NA
		Biguanides+ Sulfonylureas	6 (519)	-0.34 [-0.47, -0.22]*	44.7%
		Biguanides+ a-Glucosidase	3 (237)	-0.41 [-0.50, -0.33]*	0.0%
		Biguanides+ insulin	1 (74)	0.30 [-0.53, 1.13]	NA
	Age of patients	41< years	1 (108)	-0.44 [-0.54, -0.34]*	NA
		41–64 years	22 (1,978)	-0.34 [-0.40, -0.28]*	61.6%
		>65 years	2 (120)	-0.54 [-0.79, -0.30]*	0.0%
	FBG level at baseline	8-10mmol/L	18 (1,569)	-0.34 [-0.42, -0.25]*	76.5%
		≥10mmol/L	6 (523)	-0.39 [-0.45, -0.34]*	0.0%
	TCM syndrome differentiation	qi and yin deficiency	13 (1,021)	-0.41 [-0.52, -0.29]*	79%
		yin deficiency and excessive heat	2 (246)	-0.35 [-0.56, -0.14]*	71.2%
		Spleen deficiency	5 (409)	-0.33 [-0.41, -0.24]*	0.0%
		Phlegm-damp and blood stasis	2 (132)	-0.37 [-0.45, -0.30]*	0.0%
	Disease duration	<5 years	9 (725)	-0.34 [-0.40, -0.29]*	0.0%
		≥5–10 years	9 (746)	-0.29 [-0.40, -0.18]*	64.8%
		≥10 years	3 (300)	-0.75 [-1.27, -0.22]*	89.7%
	BMI level at baseline	≥24-28kg/m^2^	4 (298)	-0.35 [-0.47, -0.23]*	0.0%
TC (mmol/L)	All studies	All studies	29 (2,502)	-0.74 [-0.96, -0.52]*	96.1%
	Risk of bias SG	Low risk of bias SG	12 (1,178)	-0.84 [-1.10, -0.59]*	93.8%
	Comparator Drug class	Biguanides	11 (1,030)	-0.66 [-1.01, -0.32] *	97.6%
		Sulfonylureas	2 (60)	-1.07 [-1.42, -0.71]*	89.1%
		Insulin	1 (60)	-0.66 [-0.95, -0.37]*	NA
		Biguanides+ Sulfonylureas	6 (519)	-0.69[-1.15, -0.23]*	73.9%
		Biguanides+ a-Glucosidase	2 (108)	-0.93 [-2.01, 0.16]	0.0%
	Age of patients	41< years	1 (108)	-1.47 [-1.85, -1.09]*	NA
		41–64 years	22 (1,909)	-0.74 [-1.00, -0.48]*	97%
		>65 years	2 (120)	-0.44 [-1.00, 0.12]	61.8%
	FBG level at baseline	8–10 mmol/L	17 (1,500)	-0.81 [-1.15, -0.47] *	97.6%
		≥10 mmol/L	6 (523)	-0.75 [-1.18, -0.32] *	76.6%
	TCM syndrome differentiation	qi and yin deficiency	13 (1021)	-0.78 [-1.11, -0.45] *	96%
		Yin deficiency and excessive heat	2 (246)	1.36 [-1.48, -1.24]*	0.0%
		Spleen deficiency	5 (409)	-0.68 [-1.08, -0.29]*	93.7%
	Disease duration	<5 years	3 (251)	-0.58 [-0.74, -0.43]*	51.9%
		≥10 years	1 (60)	-0.42 [-0.81, -0.03]*	NA
	BMI level at baseline	≥24-28kg/m^2^	4 (289)	-0.19 [-0.43, 0.06]	53.6%
LDL (mmol/L)	All	All studies	24 (2,102)	-0.60 [-0.83, -0.37]*	97.8%
	Risk of bias SG	Low risk of bias SG	10 (990)	-0.73 [-1.09, -0.36]*	98.1%
	Comparator Drug class	Biguanides	11(1,016)	-0.64 [-1.00, -0.30]*	97.8%
		Insulin	1 (60)	-1.28 [-1.96, -0.60]*	NA
		Biguanides+ Sulfonylureas	6 (519)	-0.57 [-0.89, -0.24]*	91.3%
	Age of patients	41–64 years	19 (1,769)	-0.58 [-0.83, -0.34]*	97.9%
		>65 years	2 (120)	-0.90 [-1.54, -0.26]*	61.3%
	FBG level at baseline	8–10 mmol/L	16 (1,402)	-0.70 [-1.00, -0.40]*	98.3%
		≥10 mmol/L	4 (355)	-0.22 [-0.52, 0.09]	87.5%
	TCM syndrome differentiation	qi and yin deficiency	10 (827)	-0.67 [-1.06, -0.28]*	98.2%
		yin deficiency and excessive heat	1 (138)	-1.42 [-1.53, -1.31]*	NA
		Spleen deficiency	3 (251)	-0.42 [-0.65, -0.19]*	69.8%
	Disease duration	<5 years	8 (659)	-0.77 [-1.18, -0.37]*	98.4%
		≥5–10 years	7 (612)	-0.70 [-1.15, -0.26]*	97.8%
		≥10 years	3 (300)	-0.74 [-1.27, -0.21]*	77.1%
	BMI level at baseline	≥24–28 kg/m^2^	3 (215)	-0.33 [-0.50, -0.16]*	51.6%
HDL (mmol/L)	All	All studies	23 (2,014)	0.17 [0.07, 0.27]*	96.6%
	Risk of bias SG	Low risk of bias SG	9 (930)	0.15 [-0.08, 0.38]	98%
	Comparator Drug class	Biguanides	10 (956)	0.32 [0.23, 0.42]*	92.5%
		Insulin	1 (60)	-0.07 [-0.27, 0.13]	NA
		Biguanides+ Sulfonylureas	5 (399)	0.12 [-0.06, 0.31]	81.0%
		Biguanides+ a-Glucosidase	1(60)	-0.08 [-0.18, 0.02]	NA
	Age of patients	41–64 years	17 (1,589)	0.16 [0.03, 0.28]	97.4%
		>65 years	2 (120)	0.18 [-0.31, 0.67]	0.0%
	FBG level at baseline	8–10 mmol/L	15 (1,314)	0.11 [-0.20, 0.24]	95.7%
		≥10mmol/L	4 (355)	0.16 [-0.05, 0.38]	91.7%
	TCM syndrome differentiation	qi and yin deficiency	9 (707)	0.06 [-0.13, 0.25]	97.1%
		yin deficiency and excessive heat	1 (138)	0.27 [0.12, 0.42]*	NA
		Spleen deficiency	4 (343)	0.28 [-0.02, 0.57]	96.3%
	Disease duration	<5 years	9 (719)	0.26 [0.15, 0.37]*	92.8%
		≥5–10 years	5 (432)	0.04 [-0.30, 0.38]	98.6%
		≥10 years	3 (300)	0.28 [-0.08, 0.64]	87.9%
	BMI level at baseline	≥24-28kg/m^2^	3 (215)	0.13 [-0.08, 0.34]	89.7%
FINS (mU/Lor μIU/mL)	All studies	All studies	21(1,883)	-1.29 [-1.83, -0.75]*	96.6%
	Risk of bias SG	Low risk of bias SG	6 (498)	-2.41 [-4.32–0.49]*	97.4%
	Comparator drug class	Biguanides	9 (843)	-1.50 [-2.48, -0.53]*	96%
		Sulfonylureas	2 (120)	-1.60 [-1.89, -1.32]*	0.0%
		Insulin	2 (244)	-1.33 [-3.23, 0.57]*	97.9%
		Biguanides+ Sulfonylureas	3 (223)	-0.98 [-3.12, 1.16]	52.8%
		Biguanides+ a-Glucosidase	2 (132)	-2.01 [-2.64, -1.38]*	0.0%
	Age of patients	41–64 years	19 (1,751)	-1.26 [-1.85, -0.67]*	96.5%
	FBG level at baseline	8-10mmol/L	11 (901)	-1.97 [-3.63, -0.32]*	97.2%
		≥10mmol/L	7 (731)	-0.23 [-0.56, 0.09]	73%
	TCM syndrome differentiation	qi and yin deficiency	6 (486)	-1.02 [-1.87, -0.16]*	95.8%
		Phlegm-dampness and blood stasis	2 (204)	-2.40 [-6.5, 1.64]	98%
		yin deficiency and excessive heat	2 (198)	-4.93 [-11.36, 1.50]*	95.7%
		Spleen deficiency	6 (486)	-0.42 [-2.55, 1.72]*	98.4%
	Disease duration	<5years	8 (648)	-2.27 [-3.76, -0.77]*	98.6%
		≥5–10 years	5 (445)	-1.47 [-4.62, 1.69]	96.3%
	BMI level at baseline	≥24-28kg/m^2^	2 (155)	-1.19 [-3.21, 0.82]*	81.8%
IR	All studies	All studies	18 (1,718)	-0.83 [-1.17, -0.49]*	98.1%
	Risk of bias SG	Low risk of bias SG	6 (599)	-0.93 [-1.47, -0.40]*	94.7%
	Comparator drug class	Biguanides	7 (710)	-0.80 [-1.21, -0.40]*	95.3%
		Sulfonylureas	1 (60)	-1.06 [-1.88, -0.24]*	NA
		Insulin	3 (334)	-0.80 [-1.37, -0.23]*	97.2%
		Biguanides + a-Glucosidase	2 (132)	-0.84 [-1.20, -0.48]*	0.0%
		Biguanides + Sulfonylureas	1(73)	-1.03 [-2.31, 0.25]	NA
	FBG level at baseline	8-10mmol/L	14 (1,303)	-0.81 [-1.21, -0.41]*	94.5%
		≥10mmol/L	3 (307)	-0.92 [-2.10, 0.26]	97.7%
	TCM syndrome differentiation	Phlegm-dampness and blood stasis	2 (204)	-1.55 [-1.77, -1.33]*	0.0%
		Spleen deficiency	4 (337)	-0.50 [-1.53, 0.54]	98.4%
		qi and yin deficiency	7 (688)	-0.70 [-0.98, -0.42]*	92.7%
		yin deficiency and excessive heat	2 (198)	-1.17 [-1.80, -0.54]*	0.0%
	Disease duration	<5 years	7 (591)	-0.82 [-0.97, -0.68]*	0.0%
		≥5–10 years	7 (725)	-0.74 [-1.32, -0.61]*	99.2%
	BMI level at baseline	≥24-28kg/m^2^	2 (155)	-0.93[-1.24,-0.62]*	0.0%
IS	All studies	All studies	7 (575)	0.52 [0.23, 0.80]*	95.3%
	Risk of bias SG	Low risk of bias SG	1 (66)	1.16 [1.04, 1.28]*	NA
	Comparator drug class	Biguanides	2 (176)	0.29 [-0.02, 0.61]	65%
		Sulfonylureas	1 (60)	0.21 [0.06, 0.36]*	NA
		Insulin	1 (144)	0.56 [0.47, 0.66]*	NA
		Biguanides+ Sulfonylureas	2 (129)	0.50 [0.36, 0.63]*	0%
	FBG level at baseline	8-10mmol/L	3(182)	0.65 [-0.09, 1.39]	97.8%
		≥10mmol/L	4 (393)	0.44 [0.27, 0.60]*	73.3%
	TCM syndrome differentiation	Qi and yin deficiency	1 (80)	0.15 [-0.07, 0.37]	NA
		Phlegm-dampness and blood stasis	1 (144)	0.56 [0.47, 0.66]*	NA
		yin deficiency and excessive heat	1 (60)	0.21[0.06, 0.36]*	NA
		Spleen deficiency	1 (66)	1.16 [1.04, 1.28]*	NA
	Disease duration	<5 years	3 (182)	0.65 [-0.09, 1.39]	97.8%
		≥5–10 years	1 (96)	0.47 [0.17, 0.77]*	NA
BMI (kg/m^2^)	All studies	All studies	7 (458)	-0.08 [-1.17, 1.01]	89.7%
	Risk of bias SG	Low risk of bias SG	3 (174)	-0.66 [-1.49, 0.16]	31.4%
	Comparator drug classes	Biguanides	4 (289)	0.43 [-1.14, 2.00]	91.3%
		Insulin	1 (40)	-1.62 [-3.55, 0.31]	NA
		Biguanides+ a-Glucosidase	2 (129)	-0.43 [-0.96, 0.10]	0%
	FBG level at baseline	8–10 mmol/L	5 (344)	0.18 [-1.25, 1.60]	91.9%
	TCM syndrome differentiation	qi and yin deficiency	3 (229)	0.92 [-0.76, 2.60]	91.3%
		Spleen deficiency	2 (100)	-0.65 [-1.61, 0.32]	29.2%

Note

* Statistically significant difference between groups

Abbreviations: CI, confidence interval; CM, Chinese medicine; FBG, fasting blood glucose; 2hPG, 2-hour postprandial blood glucose; HbA1c, hemoglobin A1c; TG, triglyceride; TC, total cholesterol; LDL, low density lipoprotein; HDL, high-density lipoprotein; FINS, fasting insulin; IR, insulin resistance; IS, insulin resistance index; BMI, body mass index; MD, mean difference; SG, sequence generation.

*3*.*3*.*1*.*2*. *2-hour Postprandial blood glucose (2hPG)*. Sixty-two RCTs including 5,518 participants assessed the effects of CHM plus hypoglycaemic agents compared to hypoglycaemic agents alone [18–22, 25–33, 35–50, 52–54, 56–59, 61–67, 69–71, 73–75, 77–80, 82–86, 88–90]. Treatment duration ranged from 4 to 25 weeks.

**Fig 3:**
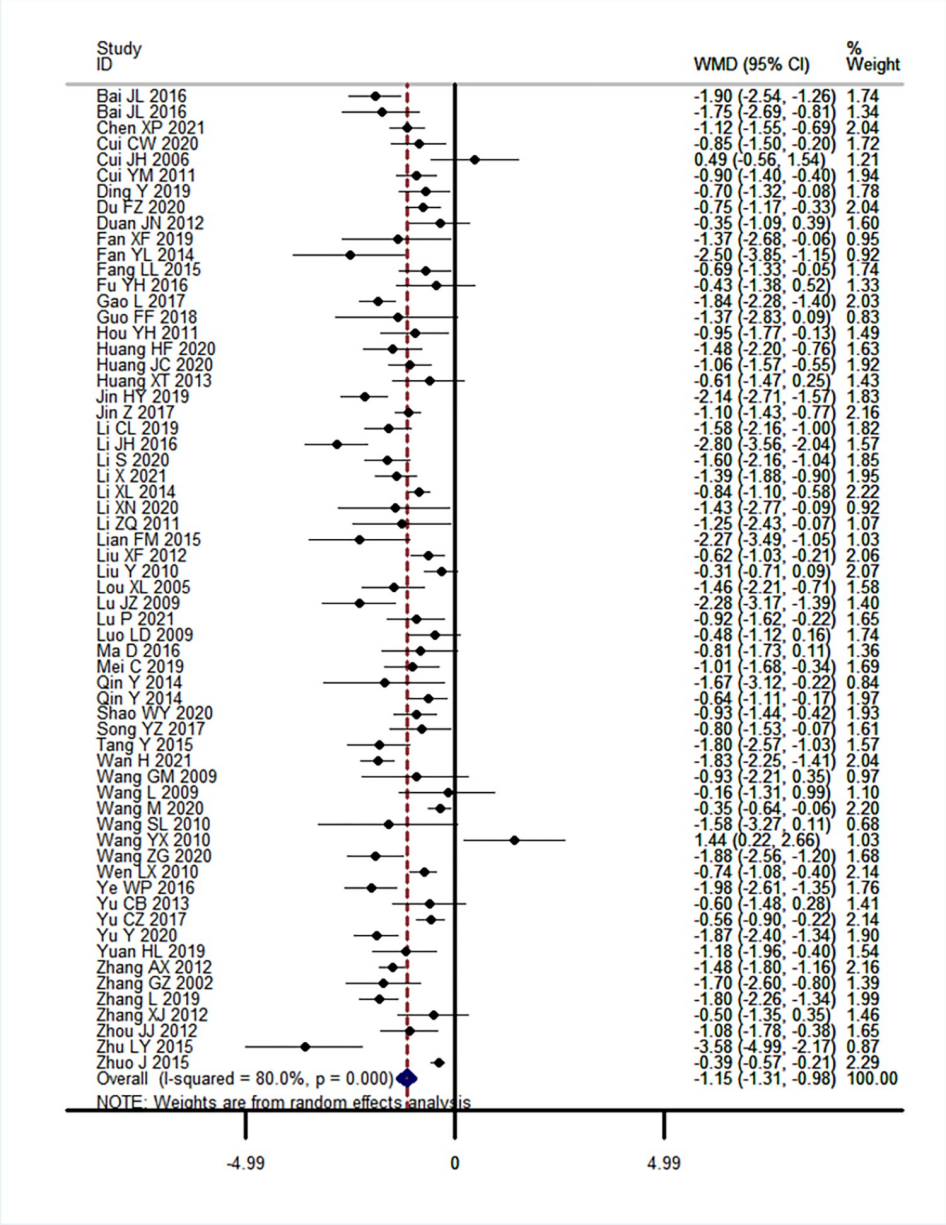
Forest plot of 2-hour postprandial blood glucose level at end of treatment, *Fuling* formulae plus hypoglycaemic agents versus hypoglycaemic agents alone, results of meta-analysis.

Meta-analysis results showed that as integrative medicine, CHM plus hypoglycaemic agents was superior to hypoglycaemic agents alone at reducing 2hPG levels at the end of treatment (MD-1.14 [-1.31, -0.98], I^2^ = 80%, *P* = 0.00, Fig 3). Meta-regression analysis found that different comparator drugs can be the possible cause of heterogeneity for this outcome (*t* = 2.08, *P =* 0.04). Sensitivity analysis by removing outlier studies (20, 57, 71) did not change the overall meta-analysis results (MD -1.17 [-1.33, -1.01]; I^2^ = 78.5% *P* = 0.00). Egger’s test demonstrated evident publication bias (*t* = 3.08, *P =* 0.00) and funnel plot showed asymmetry (S4 Fig in S1 File).

There was slight reduction in heterogeneity with studies with low risk of bias for sequence generation (27 studies, n = 2,366, MD-1.11 [-1.34, -0.87]; I^2^ = 74.3%, *P* = 0.00) (Table 3).

Grouping of studies using metformin produced the biggest pool with 27 RCTs and 2,662 participants. The effect on 2hPG is similar to the overall result with reduced heterogeneity (MD-1.29 [-1.52, -1.06]; I^2^ = 69.8%, *P* = 0.00). Combinations of different drug classes also showed significant differences between groups, with biguanides plus a-Glucosidase showing no heterogeneity. Additional subgroup analyses based on patient age groups, baseline levels of FBG, disease duration and baseline levels of BMI showed significant differences between groups (Table 3). Subgroup analysis using TCM syndrome differentiation showed favourable results in T2DM participants with *qi* and *yin* deficiency, *yin* deficiency with heat and Spleen deficiency. Interestingly, treatment duration of more than 6 months did not produce significant differences between groups, however, the result is based on one study [69].

*3*.*3*.*1*.*3*. *Hemoglobin A1c (HbA1c)*. Fifty-eight RCTs including 5,168 participants were included [18–23, 25–32, 36, 37, 39–44, 46–49, 51–54, 57, 58, 61–65, 67–70, 73–79, 81–90], treatment duration ranged from 4 to 24 weeks.

**Fig 4:**
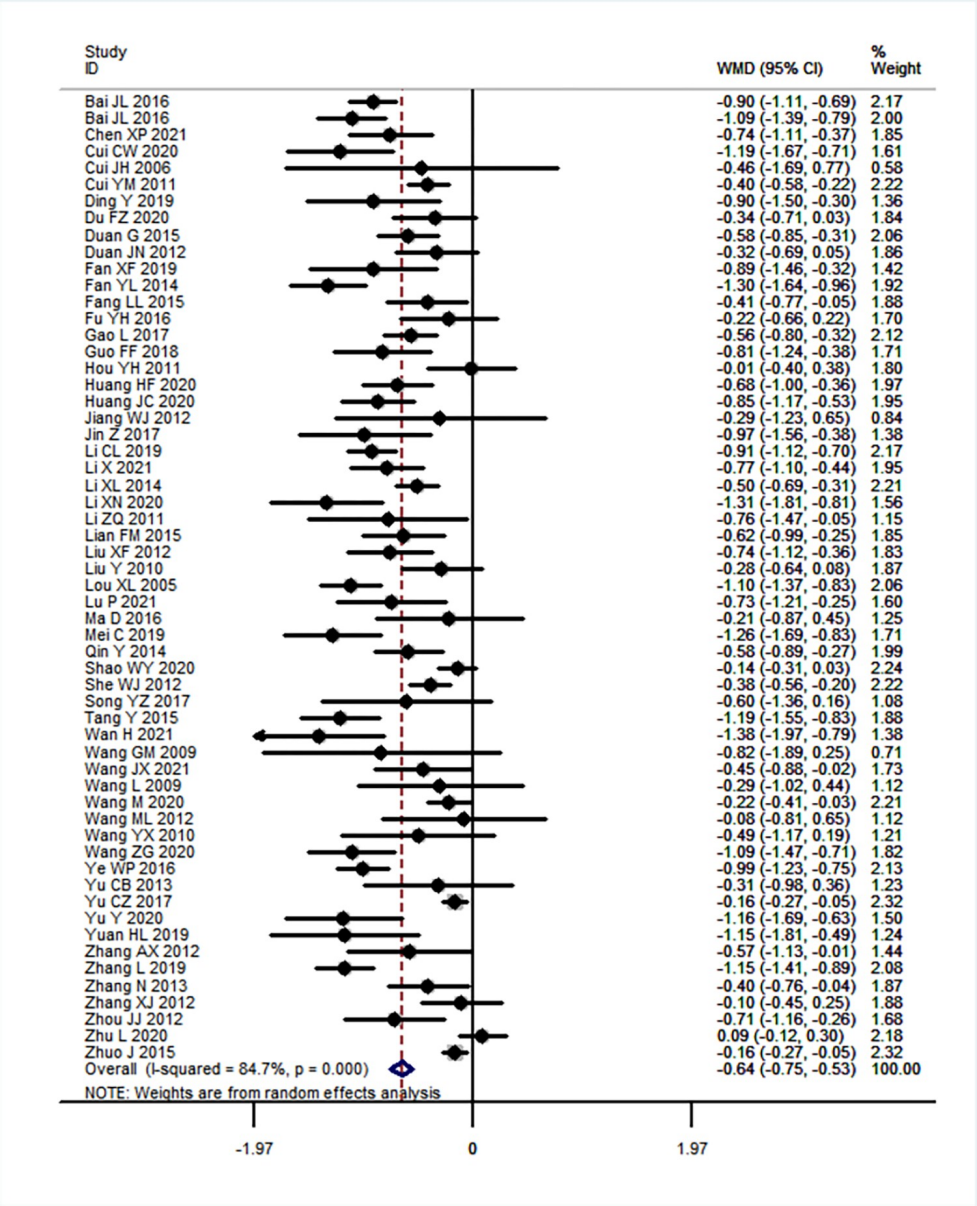
Forest plot of hemoglobin A1c level at end of treatment, *Fuling* formulae plus hypoglycaemic agents versus hypoglycaemic agents alone, results of meta-analysis.

CHM plus hypoglycaemic agents was superior to hypoglycaemic agents alone (MD-0.64 [-0.75, -0.53]; I^2^ = 84.7%, *P* = 0.00, Fig 4). Meta-regression analysis identified that the use of TCM syndrome differentiation (*t* = 3.09, P = 0.003) and sequence generation (*t* = -2.57, P = 0.01) may be a source of heterogeneity. Subgroup analysis with *qi* and *yin* deficiency reduced I^2^ from 84.7% to 64.9%. Egger’s test demonstrated there was evident of publication bias (*t* = -3.96, *P =* 0.00), asymmetry in the funnel plot was seen (S5 Fig in S1 File).

Studies with low risk of bias for sequence generation had similar results with slightly reduced heterogeneity (24 studies, n = 2,168; MD-0.54 [-0.70, -0.39], I^2^ = 78.7%, *P =* 0.00). Heterogeneity was also reduced in subgroup analysis where biguanides, sulfonylureas, or insulins were used alone (Table 3). In studies that used biguanides, the results indicated that the value of HbA_1c_ reduced in people receiving CHM plus biguanides compared to biguanides alone (MD-0.73 [-0.90, -0.56], I^2^ = 65.4%, *P =* 0.00). In older participants, HbA_1c_ values did not show a significant difference between groups. Other subgroup analyses based on comparator drug class, baseline levels of FBG, treatment duration, and disease duration showed significant differences between groups (Table 3). Subgroup analyses based on TCM syndrome differentiation showed favourable results in T2DM participants with *qi* and *yin* deficiency, phlegm-dampness and blood stasis, and Spleen deficiency. In six studies that included overweight people with diabetes, results showed improvement in HbA_1c_ values at the end of the treatment with low heterogeneity (n = 418; -0.39 [-0.57, -0.22], I^2^ = 32.8%, *P =* 0.00).

#### 3.3.2. Blood lipid metabolism indicators

*3*.*3*.*2*.*1*. *Total cholesterol (TG)*. Thirty RCTs including 2,571 participants assessed the effects of CHM plus hypoglycaemic agents versus hypoglycaemic agents alone [21, 22, 25, 26, 28–30, 32, 34, 36, 38, 42, 43, 46–48, 51, 53, 59, 65–68, 74–76, 78, 80, 83, 90]. All studies used the same hypoglycaemic agents in both groups, including metformin, glibenclamide, glipizide, glimepiride, acarbose and insulin. Treatment duration ranged from 2 to 12 weeks.

Meta-analyses showed that CHM in addition to hypoglycaemic agents was superior to hypoglycaemic agents alone at reducing TG levels in T2DM patients (MD-0.34 [-0.40, -0.29], I^2^ = 69.7%, *P* = 0.00) (S6 Fig in S1 File). Source of heterogeneity could not be identified with meta-regression analysis. Sensitivity analysis by removing outlier studies (22, 90) produced similar results to the overall meta-analysis (MD -0.33 [-0.38, -0.28]; I^2^ = 61.7% *P* = 0.00). No publication bias was detected by Egger’s test (*t =* 0.36, *P* = 0.72) nor on the funnel plot (S7 Fig in S1 File).

Meta-analysis of studies assessed as low risk for sequence generation produced a similar result with increased heterogeneity (12 RCTs, 1,178 participants, MD -0.30 [-0.39, -0.21]; I^2^ = 75.8%, *P =* 0.00). Combined use of CHM with biguanides, sulfonylureas, insulin, biguanides plus sulfonylureas and biguanides plus a-Glucosidase produced significant differences between groups, results were consistent with the overall analysis (Table 3). However, the combination of CHM to biguanides plus insulin did not produce a better result than these agents alone (Table 3). Subgroup analyses by patient age groups, FBG level at baseline, disease duration and TCM syndrome differentiation showed similar effects on TG (Table 3). In four studies that included overweight people with diabetes, results showed improvement in TG values at the end of the treatment with no heterogeneity (n = 298; -0.35 [-0.47, -0.23], I^2^ = 0%, *P =* 0.00).

*3*.*3*.*2*.*2*. *Triglyceride (TC)*. Twenty-nine RCTs including 2,502 participants assessed the effects of CHM plus hypoglycaemic agents versus hypoglycaemic agents alone [22, 25, 26, 28–30, 32, 34, 36, 38, 42, 43, 46–48, 51, 53, 59, 65–68, 74–76, 78, 80, 83, 90]. Treatment duration ranged from 2 to 12 weeks.

Meta-analyses showed that CHM in addition to hypoglycaemic agents was superior to hypoglycaemic agents alone at reducing TC levels in T2DM patients (MD -0.74 [-0.96, -0.52], I^2^ = 96.1%, *P =* 0.00) (S8 Fig in S1 File), however heterogeneity was high. When one outlier RCT was excluded from the analysis [22, 66, 90], the results remained similar (MD -0.76 [-0.99, -0.54]; I^2^ = 96.1%, *P =* 0.00). Publication bias was detected by Egger’s test (*t =* -2.11, *P* = 0.04) and observed on the funnel plot (S9 Fig in S1 File).

Meta-analysis of studies assessed as low risk of bias for sequence generation showed similar results with high heterogeneity (12 RCTs, 1,178 participants, MD-0.84 [-1.10, -0.59]; I^2^ = 93.8%, *P =* 0.00).

Subgroup analysis showed that the combination of CHM with biguanides and a-Glucosidase was not superior at reducing TC levels (Table 3). Studies with patient’s age groups of less than 40 years old and those that are 41–64 years showed a significant difference between the two groups (Table 3) but not in the 2 studies with older participants (n = 120, MD-0.44 [-1.00, 0.12]; I^2^ = 61.8%). Subgroup analyses by FBG level at baseline showed similar effects on TC. Analysis on studies that provided TCM syndrome information showed that integrative medicine was superior to hypoglycaemic agents alone in patients with *qi* and *yin* deficiency and *yin* dificiency with excessive heat and Spleen deficiency (Table 3). Subgroup analysis based on disease duration showed significant differences between groups. In studies with BMI levels indicating overweight patients at baseline showed no difference between groups (Table 3).

*3*.*3*.*2*.*3*. *Low Density Lipoprotein (LDL)*. Twenty-four RCTs including 2,102 participants assessed the effects of CHM plus hypoglycaemic agents versus hypoglycaemic agents alone [22, 26, 28–30, 32, 36, 38, 42, 43, 46, 48, 51, 53, 66–68, 74, 76, 78, 80, 81, 83, 90]. Different classes of hypoglycaemic agents were used, and treatment duration ranged from 2 to 12weeks.

Meta-analysis of 25 studies showed that CHM plus hypoglycaemic agents was superior to hypoglycaemic agents alone at reducing LDL levels at the end of treatment (MD-0.60 [-0.83, -0.37]; I^2^ = 97.8%, *P =* 0.00) (S10 Fig in S1 File), however, heterogeneity was high. Ten studies were assessed as low risk of bias for sequence generation, and subgroup analysis showed similar results to the overall studies (Table 3). Meta-regression could not identify the possible source of heterogeneity. Sensitivity analysis by removing outlier studies (51) produced similar results with high heterogeneity (MD -0.64 [-0.87, -0.41]; I^2^ = 97.1% *P* = 0.00). No publication bias was detected by Egger’s test (*t =* 0.12, *P* = 0.90) and funnel plot did not show apparent asymmetry (S11 Fig in S1 File).

Subgroup analysis by drug class showed significant differences between groups when CHM is combined with different drug classes (Table 3). In studies with higher FBG levels at baseline, at the end of the treatment, there were no significant differences between groups at the end of the treatment (4 studies, n = 355; MD-0.22 [-0.52, 0.09], I^2^ = 87.5%, *P =* 0.17). Subgroup analyses by age group or BMI level at baseline showed similar effects on LDL. In three studies that included higher BMI levels, results showed improvement in LDL values at the end of the treatment with heterogeneity (n = 215; -0.33 [-0.50, -0.16], I^2^ = 51.6%, *P =* 0.00).

*3*.*3*.*2*.*4*. *High-density lipoprotein (HDL)*. Twenty-three studies including 2,014 participants [22, 26, 28–30, 32, 36, 38, 42, 43, 46, 48, 51, 53, 65, 66, 68, 74, 76, 78, 80, 83, 90]. Treatment duration ranged from 2–12 weeks.

Meta-analysis results from the overall pool showed significant differences between the CHM plus hypoglycaemic agents to hypoglycaemic agents alone (MD 0.17 [0.07, 0.27]; I^2^ = 96.6%, *P =* 0.001) (S12 Fig in S1 File). Meta-regression results did not indicate a possible source of heterogeneity. Sensitivity analysis by removing one outlier RCT [22] produced similar results to the overall analysis (MD 0.23 [0.16, 0.30] I^2^ = 91.6%, *P =* 0.00). No publication bias was detected by Egger’s test (*t =* -1.37, *P* = 0.19), funnel plot did not show asymmetry (S13 Fig in S1 File).

Subgroup analysis in studies with low risk of bias in sequence generation showed no significant difference between groups. Subgroup analysis by hypoglycaemic agent class showed varied results for different classes of drugs, there were benefits seen in the addition of CHM to biguanides but not in other hypoglycaemic agents (Table 3). In studies that provided TCM syndrome differentiation, integrative medicine was superior to hypoglycaemic agents alone in patients with *yin* dificiency with excessive heat but not those with *qi* and *yin* deficiency and Spleen deficiency (Table 3). Participants who had diabetes for less than 5 years showed a benefit in using integrative medicine compared to hypoglycaemic agents alone (9 studies, n = 719; MD 0.26 [0.15, 0.37], I^2^ = 92.8%, *P =* 0.00), but not those with a longer disease history. Subgroup analysis on other comparator drug classes, patient’s age, FBG levels at baseline and BMI level at baseline did not produce a significant difference between groups (Table 3).

#### 3.3.3. β-cell function indicators

*3*.*3*.*3*.*1*. *Fasting insulin (FINS)*. Twenty-one RCTs including 1,883 participants assessed the effects of CHM plus hypoglycaemic agents versus hypoglycaemic agents alone for FINS [18, 23, 32, 34, 41, 46, 47, 49, 53, 55, 56, 62, 63, 66, 68, 72, 78, 80, 82, 87, 88]. One study did not use the correct unit for FINS and was removed from the analysis [48]. Different classes of hypoglycaemic agents were included, and treatment duration ranged from 4 to 12 weeks.

At the end of treatment, CHM plus hypoglycaemic agents was not superior to hypoglycaemic agents alone at reducing FINS levels (MD-1.29 [-1.83, -0.75], I^2^ = 96.6%, *P =* 0.00) (S14 Fig in S1 File). Meta-regression results did not identify possible sources of heterogeneity and sensitivity analysis by removing outlier studies [47, 53, 80, 88] did not reduce heterogeneity. No publication bias was detected by Egger’s test (*t =* -1.42, *P* = 0.17) or observed in the funnel plot (S15 Fig in S1 File).

Meta-analysis of results from studies with a low risk of bias for sequence generation showed improved results from the overall result with similar heterogeneity (6 studies, n = 498, MD-2.41 [-4.32, -0.49], I^2^ = 97.4%, *P =* 0.01). Subgroup analysis by hypoglycaemic agent class showed varied results for different class of drugs. There were benefits seen in the addition of CHM to various classes of hypoglycaemic agents but when CHM is combined with biguanides and sulfonylureas (Table 3). In studies that provided TCM syndrome differentiation, integrative medicine was superior to hypoglycaemic agents alone in patients with *qi* and *yin* deficiency, *yin* dificiency with excessive heat, Spleen deficiency but not those with phlegm-dampness and blood stasis (Table 3). People who had T2DM for more than 5 years also did not show any benefits in using CHM as integrative medicine (Table 3).

*3*.*3*.*3*.*2*. *Homeostatic model assessment of insulin resistance (IR)*. Eighteen RCTs including 1,718 participants assessed the effects of CHM plus hypoglycaemic agents versus hypoglycaemic agents alone [18, 23, 32, 41, 46, 48, 53, 56, 58, 62, 63, 68, 78, 79, 82, 84, 87, 88]. Treatment duration ranged from 4 to 12 weeks.

CHM plus hypoglycaemic agents was superior to hypoglycaemic agents alone at improving IR, however heterogeneity was high (MD -0.83 [-1.17, -0.49]; I^2^ = 98.1%, *P =* 0.00) (S16 Fig in S1 File). Meta-analysis of results from studies with a low risk of bias for sequence generation showed similar results (MD -0.93 [-1.47, -0.40]; I^2^ = 94.7%, *P =* 0.001). Meta-regression results did not identify possible sources of heterogeneity. When one outlier RCT (88) was removed, the results are similar to the overall analysis ((MD -0.91 [-1.14, -0.68]; I^2^ = 94.4%, *P =* 0.00).

Asymmetry was observed in the funnel plot (S17 Fig in S1 File) however, no publication bias was detected by Egger’s test (*t =* -1.19, *P* = 0.25). There were benefits seen in the addition of CHM to various classes of hypoglycaemic agents but not when CHM is combined with biguanides and sulfonylureas (Table 3). In people with a high level of FBG at baseline, no significant differences were found between groups. People with the TCM syndrome of Spleen deficiency also showed no significant difference between groups (Table 3).

*3*.*3*.*3*.*3*. *Insulin resistance index (IS)*. Seven RCTs including 575 participants assessed the effects of CHM plus hypoglycaemic agents versus hypoglycaemic agents alone [23, 46, 47, 49, 55, 56, 74]. One study had incorrect data and was excluded from the meta-analysis(35). Treatment duration ranged from 4 weeks to 12 weeks.

Meta-analysis showed that CHM plus hypoglycaemic agents was superior to hypoglycaemic agents alone at improving IS, however heterogeneity was high (MD 0.52 [0.23, 0.80], I^2^ = 95.3%, *P =* 0.00) (S18 Fig in S1 File). When one RCT [47] was removed from the pool, heterogeneity was reduced (MD 0.4 [0.24, 0.55], I^2^ = 77.6%, *P =* 0.00). Meta-regression was not possible for this outcome.

Analysis of one study with low risk for sequence generation found no difference between groups (66 participants, MD-0.81 [-1.73, 0.11], *P =* 0.00) [47].

Subgroup analysis by drug class showed a benefit in adding CHM to sulfonylureas(56), insulin(23), TZDs [47] and biguanide combined with sulfonylureas [46, 74], but not when CHM was added to biguanides [49, 55]. Subgroup analysis based on higher FBG level at baseline (10mmol/L) showed more benefit [23, 46, 49, 55] but not in the lower subgroup (8–10 mmol/L) (47, 56, 74). In studies that provided information on TCM differentiation, meta-analyses showed a benefit in adding CHM to hypoglycaemic agents in patients with *yin* deficiency and excessive heat, Spleen deficiency or phlegm-dampness. Studies with disease duration of more than 5 years (1 RCT, n = 96) showed a significant difference between the two groups but not in those studies with less than 5 years of disease duration (3 RCTs, n = 182) (Table 3).

#### 3.3.4. Body Mass Index (BMI)

Seven RCTs including 458 participants assessed the effects of CHM plus hypoglycaemic agents versus hypoglycaemic agents alone [21, 25, 32, 52, 60, 68, 90]. Four studies used metformin, two studies used metformin plus acarbose, and one study used insulin as comparators. Treatment duration ranged from 8 to 12 weeks.

Meta-analysis results showed that CHM plus hypoglycaemic agents did not show any significant differences to hypoglycaemic agents alone at improving BMI (MD-0.08 [-1.17, 1.01]; I^2^ = 89.7%, *P* = 0.89) (S19 Fig in S1 File). Meta-analysis of studies assessed as low risk for sequence generation found no difference between groups (3 studies, 174 participants, MD-0.66 [-1.49, 0.16]; I^2^ = 31.4%, *P* = 0.12)(25, 52, 60). Subgroup analysis based on comparator drug classes, TCM syndrome differentiation, and FBG levels at baseline showed no differences between groups (Table 3).

#### 3.3.5. Adverse events (AEs)

Out of the 73 studies, 30 studies reported on AEs. Almost half of the studies (n = 13) reported that there were no AEs in both the treatment and control groups.

Seventeen RCTs reported in details what the AEs were [25, 29, 31, 35, 37, 43, 48, 53, 66, 71, 76, 77, 79, 85, 87–89]. Two studies reported on specific AEs but did not specify which group caused these events [31, 43]. One study reported on an increase in blood pressure (2 cases), insomnia (1 case) (43); one study reported on 3 cases of diarrhoea [31].

In the integrative medicine group, 63 AEs were reported. The most common AEs were gastrointestinal symptoms including a mix of gastrointestinal symptoms of poor appetite, nausea and vomiting and diarrhoea (15 cases), or individual symptoms including diarrhea (7 cases), vomiting (2 case), nausea (2 cases), nausea and vomiting (5 case). These AEs were also present: dry mouth (2 cases), epigastric discomfort (1 case), abdominal bloating (2 cases), abdominal pain (2 cases). Other AEs included hypoglycaemia (5 cases), frequent urination (3 cases), fatigue (4 cases), dizziness (3 cases), urticaria 5 cases, and three unspecified AEs.

Seventy-three AEs was reported in the hypoglycaemic drug group. Gastrointestinal symptoms were the most common AE, these include poor appetite, nausea and diarrhoea (15 cases), nausea alone (4 cases), diarrhoea alone (2 cases), abdominal pain (1 case), gastrointestinal discomfort (4 cases), stomach distention (3 cases) and abdominal bloating (8 cases). Hypoglycaemia (11 cases) was another common AE. Other AEs include fatigue (9 cases), headache (3 cases), lactic acidosis (1 case), rash (2 cases), dry mouth (3 cases), dizziness (2 cases) and 5 other AEs that were not described in detail. Four studies reported no AEs in the control group [43, 66, 76, 82].

Although the number of AEs were less in the integrative medicine group, there were no significant difference between groups (RR = 0.99 [0.93, 1.06], *P* = 0.87) (S20 Fig in S1 File).

#### 3.3.6 Assessment of quality of evidence using GRADE

An assessment of the quality of the evidence from RCTs was undertaken using GRADE. Evidence of *Fuling* formulae for T2DM were low to moderate quality (Table 4). The results showed that oral formulae containing *Fuling* may improve glycolipid metabolism and fasting insulin level.

**Table 4:** GRADE: Quality of the evidence of *Fuling* formulae for T2DM, CHM plus Hypoglycaemic agents vs. Hypoglycaemic agents.

Outcomes	№ of participants (studies)	Certainty of the evidence (GRADE)	Anticipated absolute effects	
			Risk with [Hypoglycaemic drugs]	Risk difference with [CHM plus hypoglycaemic drugs]
Fasting blood glucose (FBG) Mean treatment duration: 9.16 weeks	6,389 (71 RCTs)	⨁⨁◯◯ LOW ^a^^,^^b^	The mean fasting blood glucose was 7.24 mmol/L	MD 0.82 mmol/L lower (0.93 lower to 0.71 lower)
2-hour Postprandial blood glucose(2hPG) Mean treatment duration: 9.53 weeks	5,518 (62 RCTs)	⨁⨁◯◯ LOW ^a^^,^^b^	The mean postprandial Blood Glucose was 9.82 mmol/L	MD 1.14 mmol/L lower (1.31 lower to 0.98 lower)
Glycosylated Hemoglobin A1c (HbA1c) Mean treatment duration: 10.07 weeks	5,168 (58 RCTs)	⨁⨁◯◯ LOW ^a^^,^^b^	The mean glycosylated Hemoglobin A1c was 7.15%	MD 0.64% lower (0.75 lower to 0.53 lower)
Triglyceride (TG) Mean treatment duration: 8.64 weeks	2,571 (30 RCTs)	⨁⨁⨁⨁ HIGH	The mean triglyceride was 2.18 mmol/L	MD 0.34 mmol/L lower (0.40 lower to 0.29 lower)
Cholesterol (TC) Mean treatment duration: 8.67 weeks	2,502 (29 RCTs)	⨁⨁◯◯ LOW ^a^^,^^b^	The mean cholesterol was 5.46 mmol/L	MD 0.74 mmol/L lower (0.96 lower to 0.52 lower)
Fasting insulin (FINS) Mean treatment duration: 9.50 weeks	1,883 (21 RCTs)	⨁⨁⨁◯ MODERATE ^a^	The mean fasting insulin was 11.22 μU/ml	MD 1.29 μU/ml lower (1.83 lower to 0.75 lower)

*The risk in the intervention group (and its 95% confidence interval) is based on the assumed risk in the comparison group and the **relative effect** of the intervention (and its 95% CI). Abbreviations: CHM: Chinese herbal medicine; CI: Confidence interval; GRADE, Grading of Recommendations Assessment, Development and Evaluation; MD: Mean difference; RCTs: randomised controlled trials

Explanations

a. High statistical heterogeneity, p<0.05

b. Publication bias detected.

## 4. Discussion

### 4.1. Summary and discussion of evidence

This study presents a comprehensive and up-to-date review of the benefits of adding herbal formulae containing *Fuling* to conventional therapies in the management of T2DM. Systematic evaluation of 73 RCTs suggested that in terms of controlling blood glucose, blood lipid, and improving insulin resistance, traditional Chinese medicine formulae containing *Fuling* combined with conventional therapies do show added benefits when compared to hypoglycaemic agents alone, and can improve important outcome measures including FBG, 2hPG, HbA_1c_, TG, TC, LDL, HDL in people with T2DM.

Decline in β-cell function is a characteristic of T2DM and there is pharmacotherapy targeted at improving β-cell function for T2DM. Insulin secretagogues agents binds to the sulfonylurea receptor on the plasma membrane of pancreatic β-cell. These agents act as glucose sensor and trigger for insulin secretion [91]. Meta-analysis results from our study showed that *Fuling* formulae together with sulfonylureas and insulin can enhance improvement in β-cell function compared to hypoglycaemic agents alone, suggesting that *Fuling* formulae may have an impact on β-cell function enhancement. Further experimental studies should be carried out to elucidate the mechanisms involved in this observation.

*Fuling* formulae combined with metformin (biguanides) creates the biggest pool when we group the included studies by hypoglycaemic drug classes. For all the included outcomes, this subgroup of studies showed benefits in adding herbal formulae to metformin, suggesting that this combination may enhance insulin-mediated glucose uptake and oxidative metabolism in peripheral tissues. Experimental studies of *Fuling* compounds shows its glucose uptake function. In murine 3T3-L1 adipocytes, *Fuling* triterpenoids (1, 2, 13, 14, 15 and 16)stimulated glucose uptake in a dose-response manner [92]. In the same study, another *Fuling* triterpenoid pachymic acid also increased glucose uptake in a time and dose-dependent manner. Further, the crude extract of *Fuling* and its triterpenes, dehydrotumulosic acid and dehydrotrametenolic acid, in streptozotocin-treated mice, whose pancreatic islet cells were killed, improved insulin sensitivity by increasing insulin-mediated blood glucose reduction, in the same manner as the insulin sensitizer metformin [93].

Being overweight and obese is one of the many risk factors for T2DM. In overweight people with T2DM, *Fuling* formulae plus hypoglycaemic agents can further reduce FGB, 2hPG, HbA_1c_, TG, LDL, FINs and IR levels. *In vitro* and *in vivo* studies of *Fuling* has also shown evidence of these observed benefits. In T2DM animal model using *ob/ob* mice, oral administration of water insoluble polysaccharides (50 or 100 mg/kg body) for 4 weeks significantly reduced both fasting blood glucose and free diet blood glucose [94]. HbA1c levels was also reduced substantially, and glucose tolerance and insulin resistance also showed improvements. Water insoluble polysaccharides also decreased levels of serum TC, TG and LDL-C levels in the *ob/ob* mice compared to the vehicle control group [94]. Further examination of adipocytes showed that oral treatment with water insoluble polysaccharides prevented adipocyte hypertrophy, indicating an effect for hyperlipidaemia [94].

Heterogeneity in our meta-analysis was high, thus lowering the evidence grade. For studies that included FBG as an outcome, meta-regression identified that baseline FBG levels and reporting of TCM syndrome may be the source of heterogeneity between studies. Reporting of TCM syndrome was also a possible source for heterogeneity for HbA_1c_. Different classes of hypoglycaemic drugs were identified to be a possible source of heterogeneity for studies that included 2hPG as an outcome. Further, we performed sensitivity analysis by omitting outlier study(ies), however, this did not help reduce heterogeneity in our included outcome measures.

Considering different herb usage in different clinical trials, this could be a possible source of heterogeneity in the meta-analysis, further analysis of herb usage could be explored in future studies.

Publication bias was evident in studies that reported on FBG, 2hPG, HbA_1c_ and TC. This indicates that the available studies may not represent the entire evident base for this topic.

Adverse events in the herbal formula group are lower than the control group, suggesting a good safety profile. Taken together, Chinese herbal medicine therapy including *Fuling* can be beneficial for patients with T2DM.

### 4.2. Limitations of the current review

There are limitations with the current review. Included clinical studies presented possible methodological shortfalls. In randomised clinical trials, appropriate randomization and allocation concealment methods can reduce bias. However, only 30/73 (41%) of the included studies described the randomisation methods, and 4% of the studies described allocation concealment. The efficacy of *Fuling* formulae may be exaggerated by inappropriate randomization and allocation concealment methods.

Furthermore, overestimation of the effect can result from inadequate blinding of treatment groups. There are many barriers in implementing blinding in Chinese herbal medicine trials. It is possible for participants to identify which group they are in based on if they have been given CHM. Therefore, risk of bias exists for this domain and trial results may be compromised if the participants could determine which group they were allocated in.

Not all included studies published study protocols and this non-standardization of clinical research reports is unhelpful to the dissemination of the study results.

T2DM is a chronic and progressive disease, and those who are affected may have drastic lifestyle changes. However, outcomes related to quality of life were not reported. Also, the clinical studies had treatments between 2–24 weeks with only four studies reporting on follow-up data. This may not reflect the true nature of the disease and the long-term benefit of adding *Fuling* formulae to hypoglycaemic agents is unknown.

The systematic review was of Chinese medicine formulae containing *Fuling*. Included studies all used *Fuling* as a part of the herbal formula for T2DM. Therefore, the analysis and results of this study do not fully represent the role of *Fuling* alone in the treatment of T2DM in reducing blood sugar, lipid and improving IR.

During the review process, unintentional human errors can occur during the screening and analysis process, although strict procedures are followed.

Taken all the above limitations into consideration, it is necessary to interpret these results with caution before translating into clinical practice.

### 4.3. Implications in research

Future clinical studies should be recommended to design and report data following the items required by the Consolidated Standards of Reporting Trials (CONSORT) [95] and its extensions for herbal medicine and traditional TCM [96, 97].

Rigorous methodology is recommended when designing future clinical trials, with correct methods of sequence generation and allocation concealment. Options for herbal formulae placebo should be explored to provide an opportunity for blinding and reducing risk of bias in this domain. Protocols should be published and be registered to minimize reporting bias and increase transparency in the reporting the results.

Based on our meta-regression results, when including T2DM patients for herbal formulae studies, researchers can consider implementing TCM syndrome differentiation, this way similar groups of participants can be included whilst the same formula is given. This conforms with clinical practice of TCM and may help reduce heterogeneity in future meta-analysis of similar studies. Another observation from meta-regression is when combining RCTs for analysis, studies that use the same hypoglycaemic agents should be analysed together, as this may be another source of heterogeneity.

In terms of outcome measures, quality of life for T2DM participants is an important clinical outcome and would provide further evidence of TCM therapies for T2DM.

T2DM is a progressive and lifelong disease. Future studies may consider using longer treatment durations and a lengthier follow-up period, to reflect clinical practice and provide evidence for the long-term effects of TCM treatments using *Fuling* formulae.

## 5. Conclusion

In closing, this study presented clinical evidence and a detailed review of *Fuling* formulae for the treatment of T2DM. Systematic evaluation of RCT data suggested that for T2DM, traditional Chinese medicine formulae containing *Fuling* combined with conventional therapies do show added benefits when compared to hypoglycaemic agents alone. Poor methodology quality of trial design from some included trials were found in this review, therefore results need to be interpreted with caution. Future trials should include rigorous trial methodological trial design and follow standard reporting methods such as CONSORT. Future trials should also consider having longer follow up periods and consider quality of life measures for T2DM.

## Supporting information

S1 Checklist (DOCX)

S2 Checklist (DOCX)

S1 Table Search syntax for databases. (DOCX)

S1 File Supplementary figures S1-S20. (DOCX)

S1 Dataset (XLSX)
