# Supplementary material for: Benefits of herbal formulae containing Poria cocos (Fuling) for type 2 diabetes mellitus: A systematic review and meta-analysis
Source: PLoS One. 2022 Dec 1;17(12):e0278536. doi: 10.1371/journal.pone.0278536 (PMC9714931; doi:10.1371/journal.pone.0278536)
Supplement: S1 Table — (DOCX) [file pone.0278536.s003.docx]

Supplementary Table 1. Search syntax for databases

| **Pubmed** | |
| --- | --- |
| Chinese herbal medicine | Traditional Chinese Medicine OR Chinese Traditional Medicine OR Chinese Herbal Drugs OR Chinese Drugs, Plant OR Medicine, Traditional OR Ethnopharmacology OR Ethnomedicine OR Ethnobotany OR Medicine, Kampo OR Kanpo OR TCM OR Medicine, Ayurvedic OR Phytotherapy OR Herbology OR Plants, Medicinal OR Plant Preparation OR Plant Extract OR Plants, Medicine OR Materia Medica OR Single Prescription OR Chinese Medicine Herb OR Herbal Medicine OR Herbs |
| Type 2 Diabetes mellitus | Diabetes Mellitus, Noninsulin-Dependent OR Diabetes Mellitus, Ketosis-Resistant OR Diabetes Mellitus, Non Insulin Dependent OR Diabetes Mellitus, Stable OR Diabetes Mellitus, Type II OR NIDDM OR Diabetes Mellitus, Noninsulin Dependent OR Diabetes Mellitus, Maturity-Onset OR MODY OR Diabetes Mellitus, Slow-Onset OR Type 2 Diabetes Mellitus OR Maturity-Onset Diabetes OR Type 2 Diabetes OR Diabetes, Type 2 OR Diabetes Mellitus, Adult-Onset |
| Randomised controlled trial | "randomized controlled trial"[pt] OR "controlled clinical trial"[pt] OR "randomized"[tiab] OR "placebo"[tiab] OR "drug therapy"[sh] OR "randomly"[tiab] OR "trial"[tiab] OR "groups"[tiab] |
| **Excerpta Medica Database (Embase)** | |
| Chinese herbal medicine | Traditional Chinese Medicine OR Chinese Traditional Medicine OR Chinese Herbal Drugs OR Chinese Drugs, Plant OR Medicine, Traditional OR Ethnopharmacology OR Ethnomedicine OR Ethnobotany OR Medicine, Kampo OR Kanpo OR TCM OR Medicine, Ayurvedic OR Phytotherapy OR Herbology OR Plants, Medicinal OR Plant Preparation OR Plant Extract OR Plants, Medicine OR Materia Medica OR Single Prescription OR Chinese Medicine Herb OR Herbal Medicine OR Herbs |
| Type 2 Diabetes mellitus | non insulin Dependent Diabetes Mellitus OR type 2 diabetes |
| Randomised controlled trial | randomized controlled trial or controlled clinical trial or randomized or placebo or drug therapy or randomly or trial or groups |
| **Cochrane Central Register of Controlled Trials (CENTRAL)** | |
| Chinese herbal medicine | Traditional Chinese Medicine OR Chinese Traditional Medicine OR Chinese Herbal Drugs OR Chinese Drugs, Plant OR Medicine, Traditional OR Ethnopharmacology OR Ethnomedicine OR Ethnobotany OR Medicine, Kampo OR Kanpo OR TCM OR Medicine, Ayurvedic OR Phytotherapy OR Herbology OR Plants, Medicinal OR Plant Preparation OR Plant Extract OR Plants, Medicine OR Materia Medica OR Single Prescription OR Chinese Medicine Herb OR Herbal Medicine OR Herbs |
| Type 2 Diabetes mellitus | diabetes mellitus type 2 OR non insulin Dependent Diabetes Mellitus |
| **Cumulative Index of Nursing and Allied Health Literature (CINAHL)** | |
| Chinese herbal medicine | Traditional Chinese Medicine OR Chinese Traditional Medicine OR Chinese Herbal Drugs OR Chinese Drugs, Plant OR Medicine, Traditional OR Ethnopharmacology OR Ethnomedicine OR Ethnobotany OR Medicine, Kampo OR Kanpo OR TCM OR Medicine, Ayurvedic OR Phytotherapy OR Herbology OR Plants, Medicinal OR Plant Preparation OR Plant Extract OR Plants, Medicine OR Materia Medica OR Single Prescription OR Chinese Medicine Herb OR Herbal Medicine OR Herbs |
| Type 2 Diabetes mellitus | diabetes mellitus type 2 OR non insulin Dependent Diabetes Mellitus |
| Randomised controlled trial | randomized controlled trial OR controlled clinical trial OR randomized OR placebo OR drug therapy OR randomly OR trial OR groups |
| **Allied and Complementary Medicine Database (AMED)** | |
| Chinese herbal medicine | Traditional Chinese Medicine OR Chinese Traditional Medicine OR Chinese Herbal Drugs OR Chinese Drugs, Plant OR Medicine, Traditional OR Ethnopharmacology OR Ethnomedicine OR Ethnobotany OR Medicine, Kampo OR Kanpo OR TCM OR Medicine, Ayurvedic OR Phytotherapy OR Herbology OR Plants, Medicinal OR Plant Preparation OR Plant Extract OR Plants, Medicine OR Materia Medica OR Single Prescription OR Chinese Medicine Herb OR Herbal Medicine OR Herbs |
| Type 2 Diabetes mellitus | type 2 diabetes OR t2dm OR non-insulin dependent diabetes OR non insulin dependent diabetes mellitus OR type 2 diabetes mellitus |
| Randomised controlled trial | randomized controlled trial OR controlled clinical trial OR randomized OR placebo OR drug therapy OR randomly OR trial OR groups |
| **Chinese-language databases included China SinoMed Literature** | |
| Chinese herbal medicine | 中医 OR 中西医 OR 中医疗法 OR 辨病OR 辨证 OR 辨证论治 OR 辨症施治 OR 辩证 OR 汉方 OR 祖国医学 OR 传统医学 OR 传统治疗 OR 传统疗法 OR 替代医学 OR 替代治疗 OR 中国传统医学 OR 民族医药 OR 民族医学 OR 草药 OR 中草药 OR 中药 OR 中药疗法 OR 中西药 OR 传统医药 OR 中成药 OR 植物药 OR 中医治法 OR 治则 OR 中医疗法 OR 熏洗 OR 浸洗 OR 药浴 OR 洗浴 OR 外洗 OR 沐足 OR 足浴 OR 浴足 OR 灌肠 OR 熨法 OR 药熨 OR 熨药 OR 热熨 OR 热敷 OR 敷脐 OR 药枕 OR 药烘 OR 足疗 OR雾化 OR 中药外敷 OR 外敷 OR蒸熏 OR熏蒸 |
| Type 2 Diabetes mellitus | 成年型糖尿病、非酮症性糖尿病、非胰岛素依赖型糖尿病、稳定性糖尿病、Ⅱ型糖尿病、老年2型糖尿病、初诊2型糖尿病、胰岛素抵抗、糖化血红蛋白、空腹血糖 |
| Randomised controlled trial | 临床观察 OR 临床评估 OR 临床试验 OR 临床效果 OR 临床研究 OR 疗效 OR 评价研究 OR 前瞻性 OR 随访 OR 对比研究 OR 多中心 OR 随机 OR 对照 OR 病例报告 OR 病例研究 OR 病例分析 OR 病例报道 |
| **China National Knowledge Infrastructure (CNKI)** | |
| Chinese herbal medicine | 中医 OR SU%中西医 OR SU%中医疗法 OR SU%辨病 OR SU%辨证 OR SU%辨证论治 OR SU%辨症施治 OR SU%传统医学 OR SU%传统疗法 OR SU%替代医学 OR SU%民族医药 OR SU%草药 OR SU%中草药 OR SU%中药 OR SU%中西药 OR SU%传统医药 OR SU%中成药 OR SU%植物药 OR SU%中医治法 OR SU%治则 OR SU%中医疗法 OR SU%熏洗 OR SU%沐足 OR SU%足浴 |
| Type 2 Diabetes mellitus | 2型糖尿病 OR SU%成年型糖尿病 OR SU%非酮症性糖尿病 OR SU%非胰岛素依赖型糖尿病 OR SU%稳定性糖尿病 OR SU%Ⅱ型糖尿病 OR SU%老年2型糖尿病 OR SU%初诊2型糖尿病 OR SU%胰岛素抵抗 OR SU%糖化血红蛋白 OR SU%空腹血糖 OR SU%II型糖尿病 |
| Randomised controlled trial | 临床观察 OR SU%临床评估 OR SU%临床试验 OR SU%临床效果 OR SU%临床研究 OR SU%疗效 OR SU%评价研究 OR SU%前瞻性 OR SU%随访 OR SU%对比研究 OR SU%多中心 OR SU%随机 OR SU%对照 OR SU%病例报告 OR SU%病例研究 OR SU%病例分析 OR SU%病例报道 |
| **Chongqing VIP (CQVIP)** | |
| Chinese herbal medicine | M=中医+M=中西医+M=中医疗法+M=辨证+M=辨证论治+M=辨症施治+M=传统医学+M=替代医学+M=中医治法+M=中医疗法 |
| Type 2 Diabetes mellitus | M=2型糖尿病+M=成年型糖尿病+M=非酮症性糖尿病+M=非胰岛素依赖型糖尿病+M=稳定性糖尿病+M=Ⅱ型糖尿病+M=老年2型糖尿病+M=初诊2型糖尿病+M=胰岛素抵抗+M=糖化血红蛋白+M=空腹血糖+M=II型糖尿病 |
| Randomised controlled trial | 临床试验+M=临床研究+M=前瞻性+M=随访+M=多中心+M=随机+M=对照+M=病例报告+M=病例研究 |
| **Wanfang** | |
| Chinese herbal medicine | 中医 or 中西医 or 辨证论治 or 辨症施治 or 传统医学 or 替代医学 |
| Type 2 Diabetes mellitus | 2型糖尿病 or 成年型糖尿病 or 非酮症性糖尿病 or 非胰岛素依赖型糖尿病 or 稳定性糖尿病 or Ⅱ型糖尿病 or II型糖尿病 |
| Randomised controlled trial | 临床试验 or 临床研究 or 前瞻性 or 随访 or 多中心 or 随机 or 对照 or 病例报告 or 病例研究 |
