# Supplementary material for: Benefits of herbal formulae containing Poria cocos (Fuling) for type 2 diabetes mellitus: A systematic review and meta-analysis
Source: PLoS One. 2022 Dec 1;17(12):e0278536. doi: 10.1371/journal.pone.0278536 (PMC9714931; doi:10.1371/journal.pone.0278536)
Supplement: S1 File — (DOCX) [file pone.0278536.s004.docx]

Supplementary Materials

**S1 Fig. Risk of bias graph for included studies**


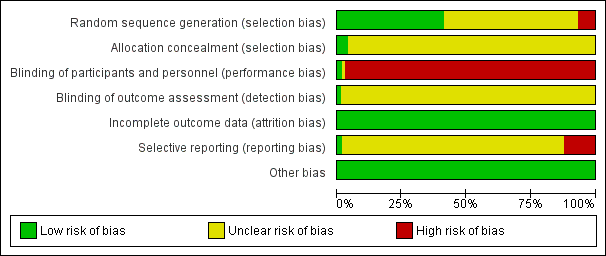


**S2. Fig. Risk of bias summary for individual studies**


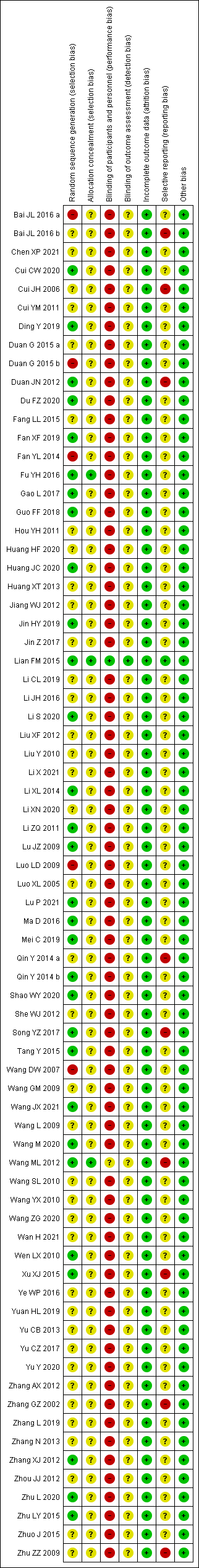


**S3 Fig. Funnel plot of *Fuling* formulae plus hypoglycaemic agents versus hypoglycaemic agents alone for fasting blood glucose**


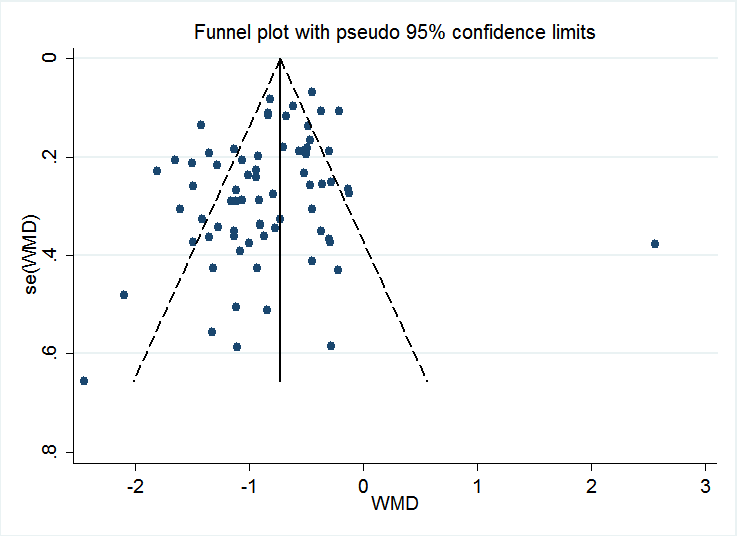


**S4 Fig. Funnel plot of *Fuling* formulae plus hypoglycaemic agents versus hypoglycaemic agents alone for 2-hour postprandial blood glucose**

**
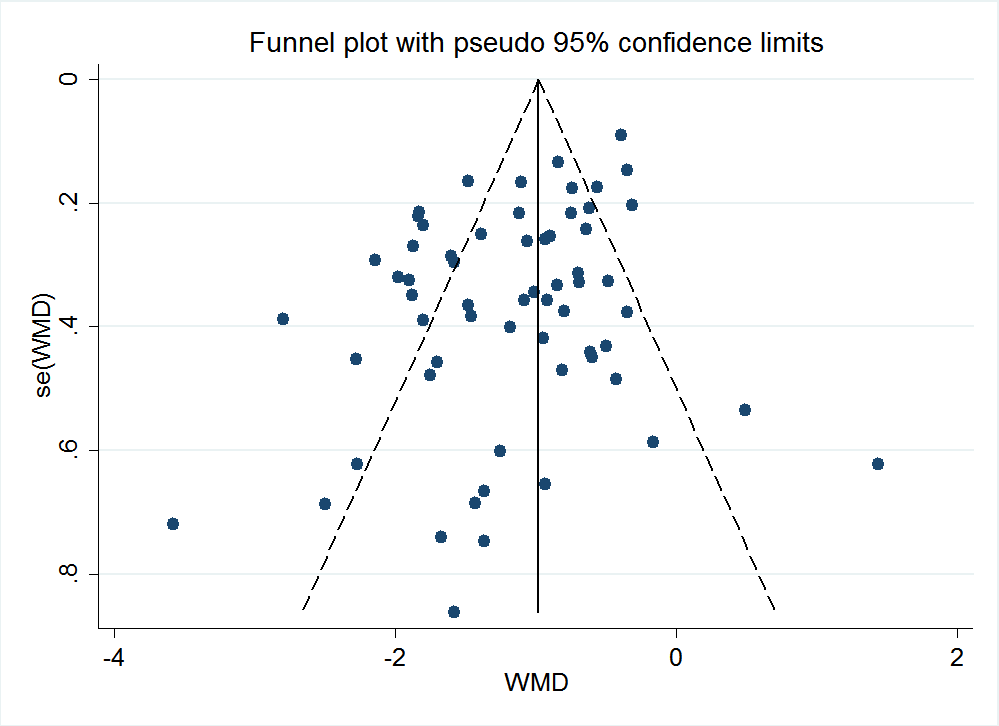
**

**S5 Fig. Funnel plot of *Fuling* formulae plus hypoglycaemic agents versus hypoglycaemic agents alone for hemoglobin A1c**

**
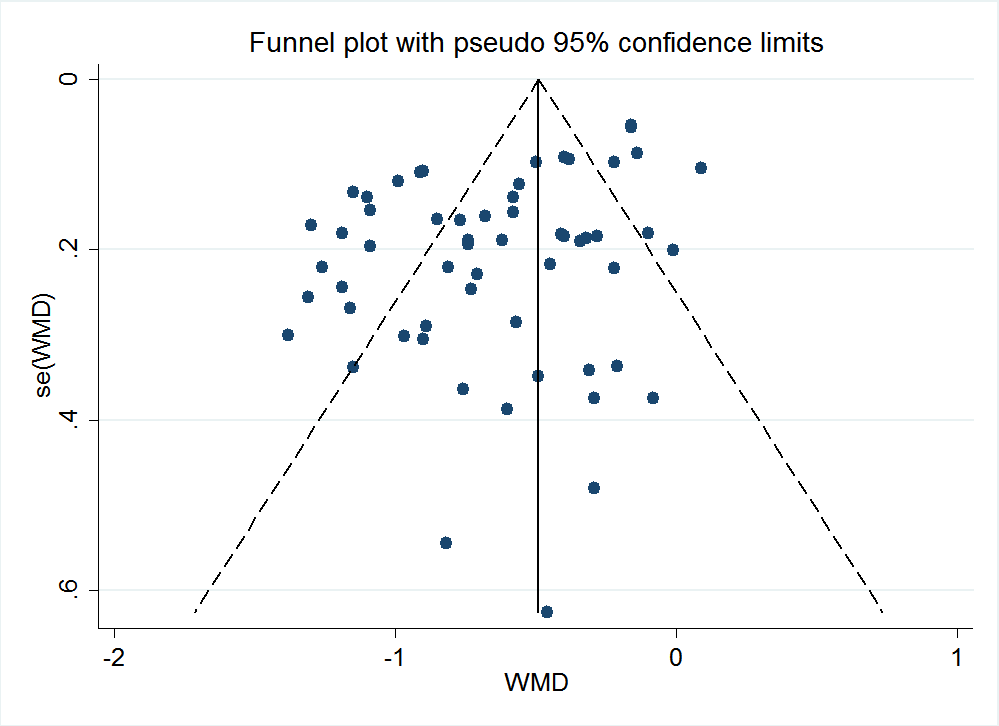
**

**S6 Fig. Forest plot of total cholesterol level at end of treatment, *Fuling* formulae plus hypoglycaemic agents versus hypoglycaemic agents alone, results of meta-analysis.**

**
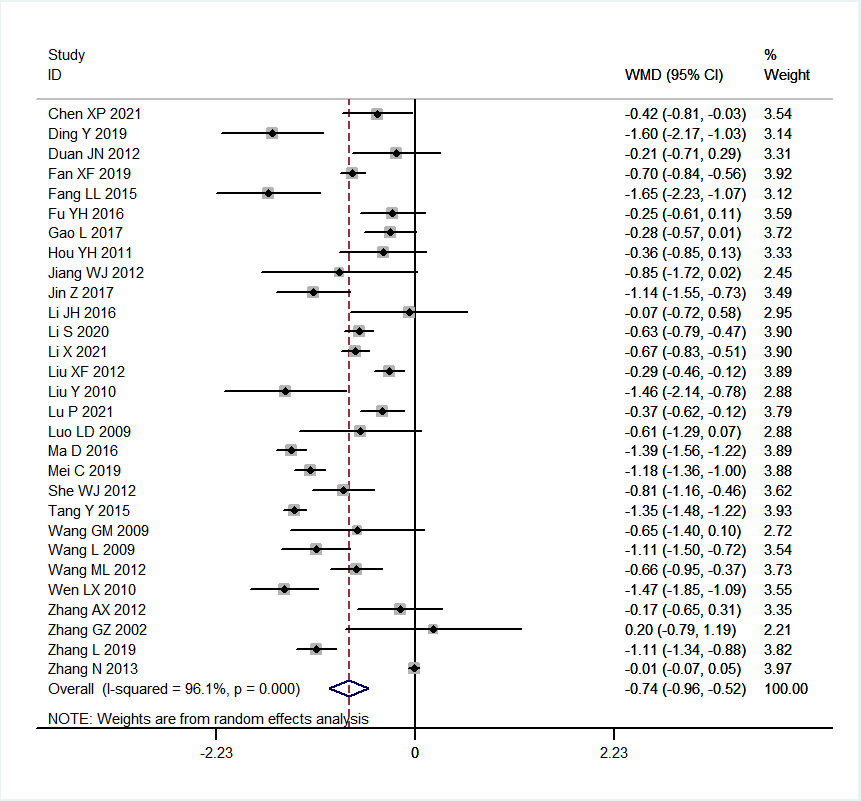
**

**S7 Fig. Funnel plot of *Fuling* formulae plus hypoglycaemic agents versus hypoglycaemic agents alone for total cholesterol**

**
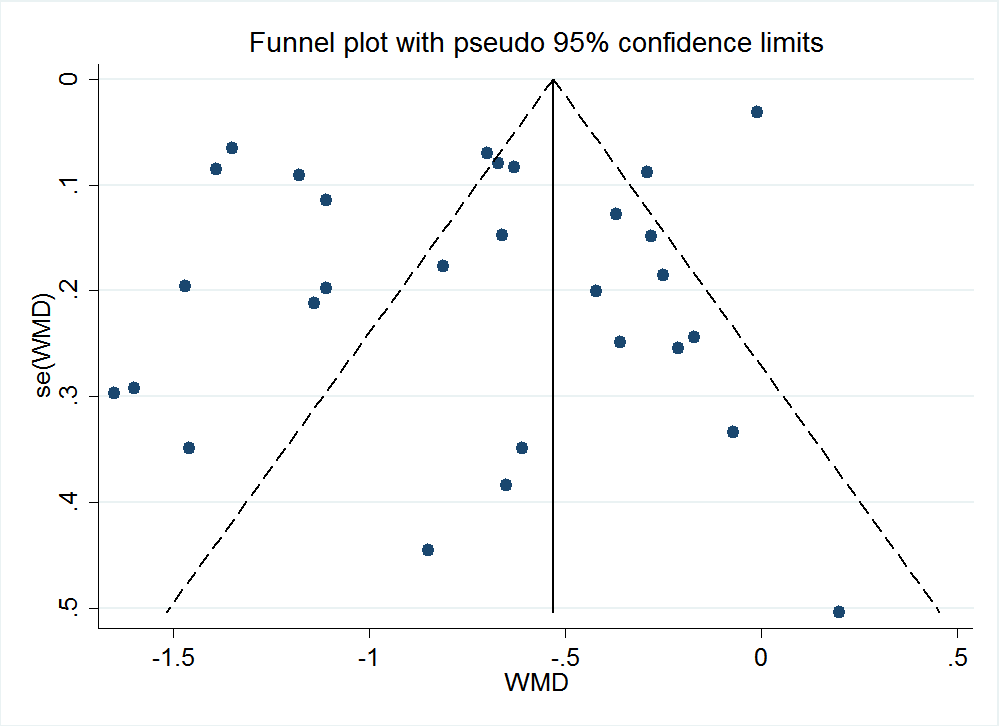
**

**S8 Fig. Forest plot of triglyceride level at end of treatment, *Fuling* formulae plus hypoglycaemic agents versus hypoglycaemic agents alone, results of meta-analysis.**


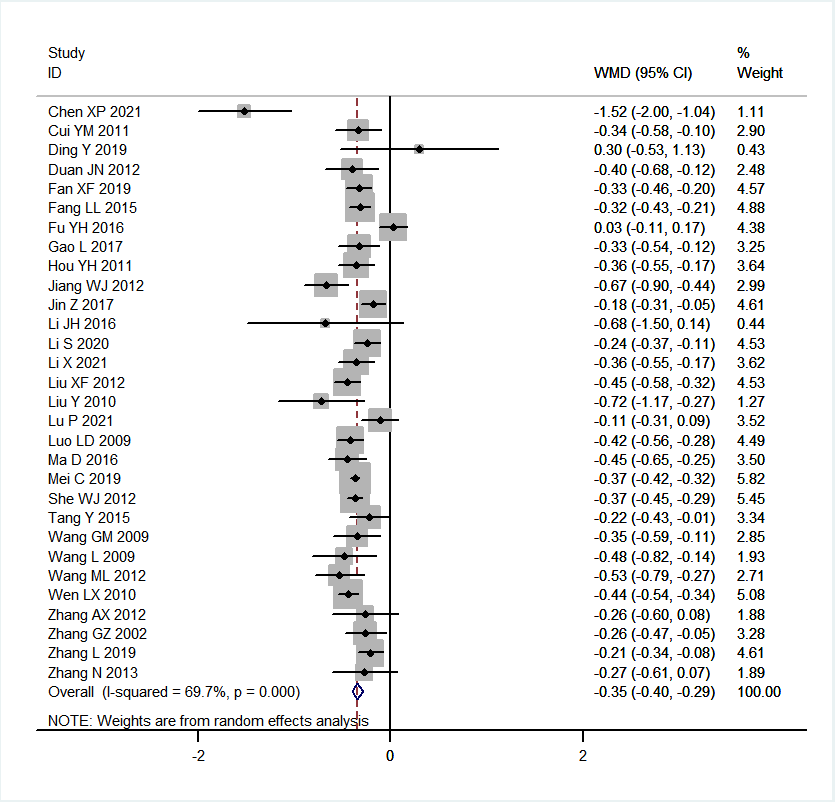


**S9 Fig. Funnel plot of *Fuling* formulae plus hypoglycaemic agents versus hypoglycaemic agents alone for triglyceride**

**
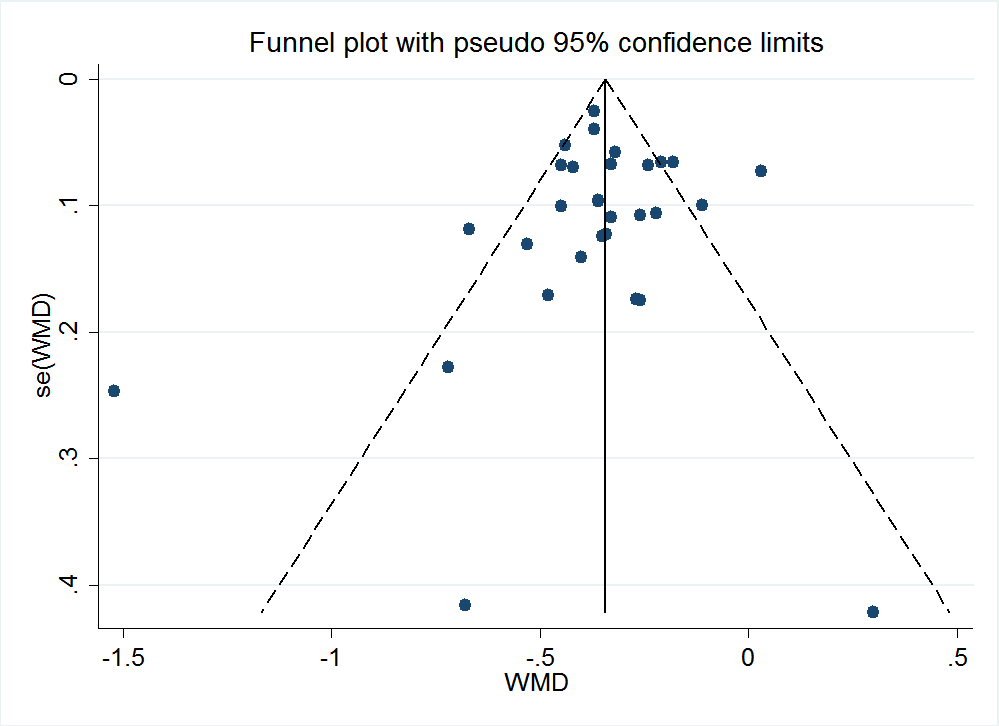
**

**S10 Fig. Forest plot of low density lipoprotein level at end of treatment, *Fuling* formulae plus hypoglycaemic agents versus hypoglycaemic agents alone, results of meta-analysis.**

**
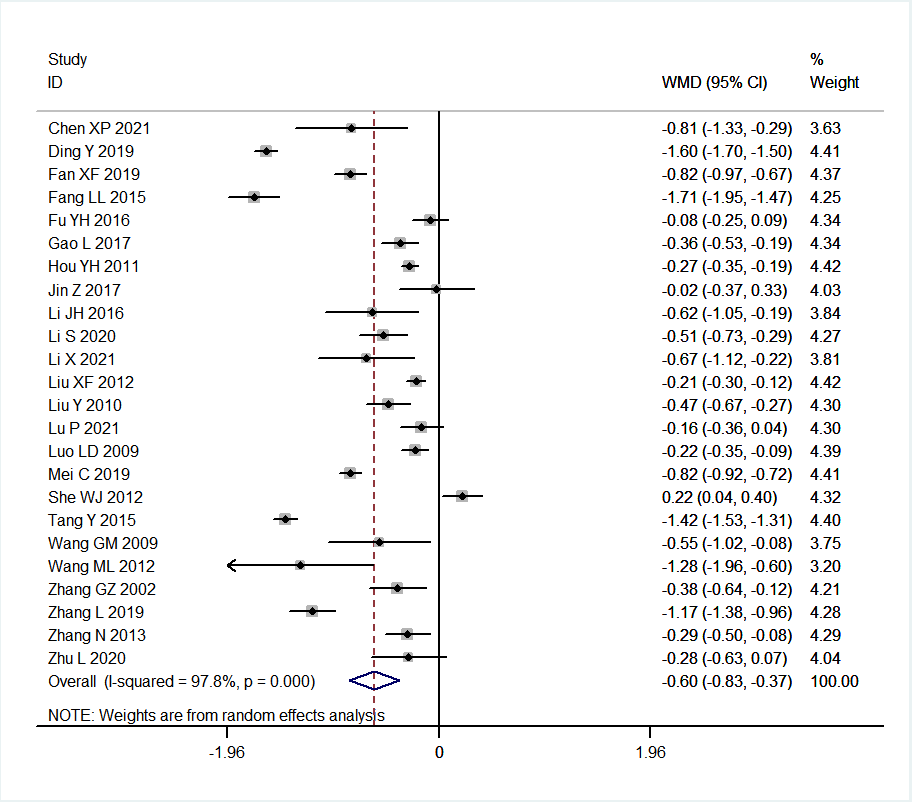
**

**S11 Fig. Funnel plot of *Fuling* formulae plus hypoglycaemic agents versus hypoglycaemic agents alone for low density lipoprotein**

**
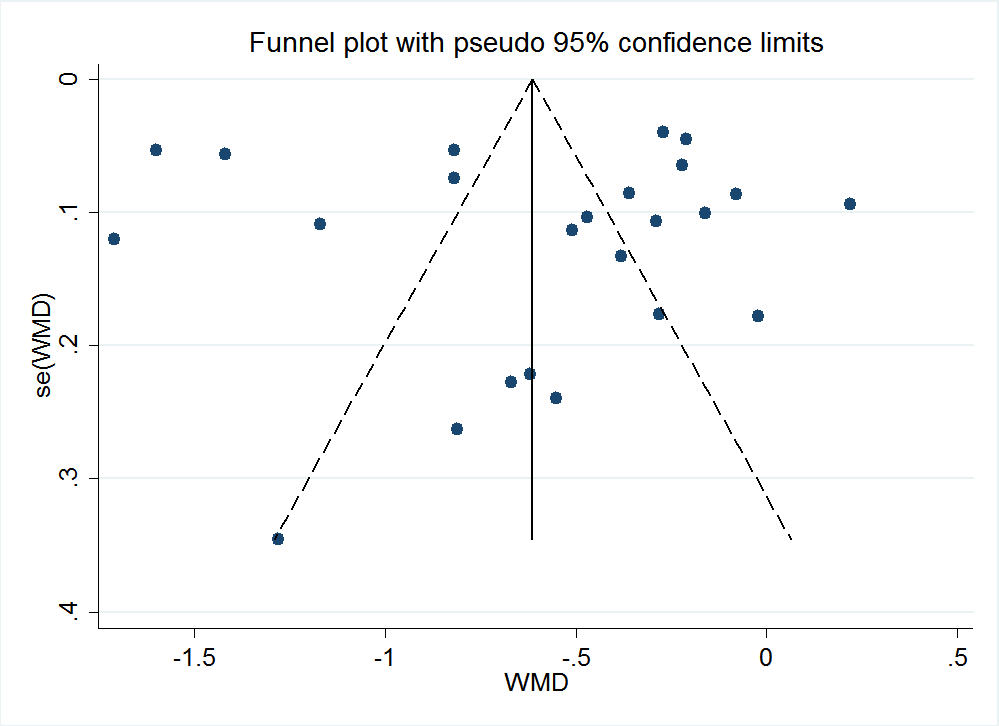
**

**S12 Fig. Forest plot of high density lipoprotein level at end of treatment, *Fuling* formulae plus hypoglycaemic agents versus hypoglycaemic agents alone, results of meta-analysis.**

**
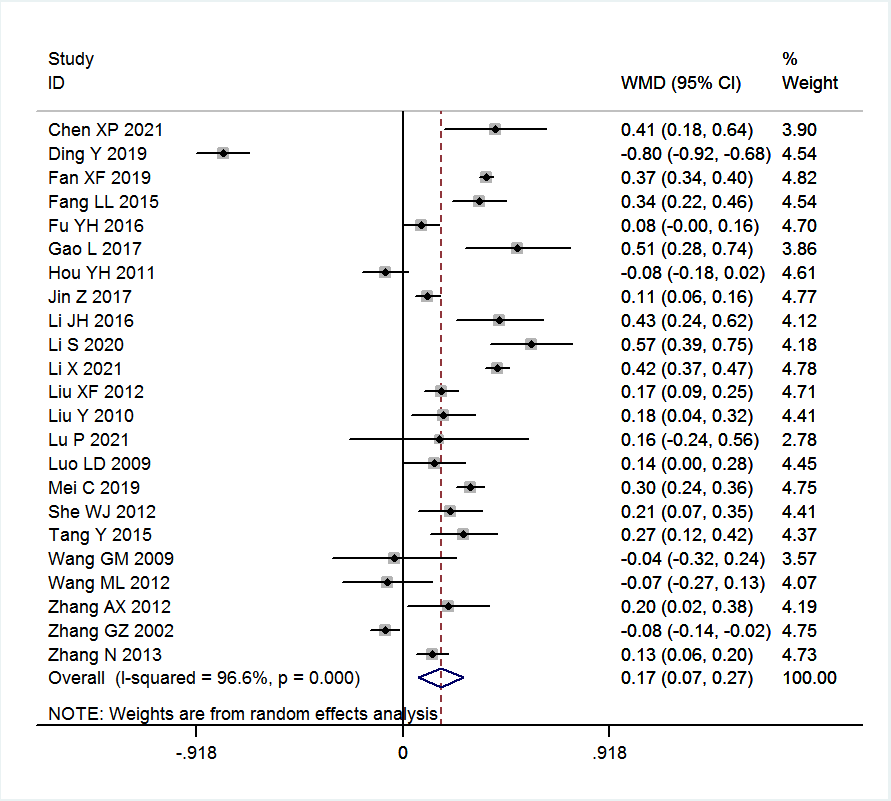
**

**S13 Fig. Funnel plot of *Fuling* formulae plus hypoglycaemic agents versus hypoglycaemic agents alone for high density lipoprotein**

**
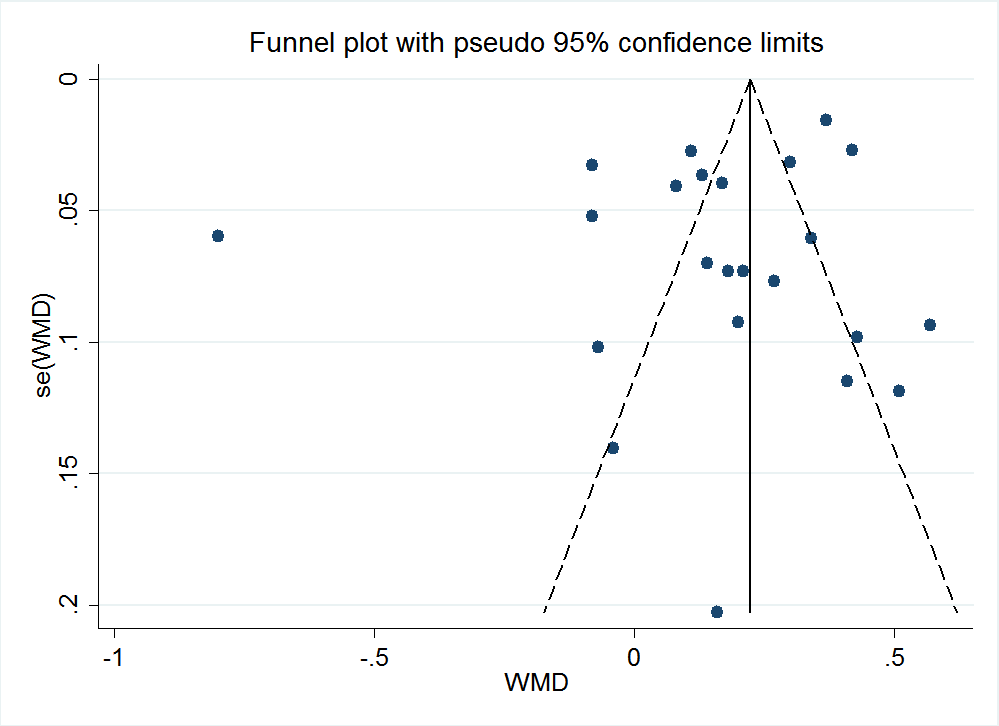
**

**S14 Fig. Forest plot of fasting insulin level at end of treatment, *Fuling* formulae plus hypoglycaemic agents versus hypoglycaemic agents alone, results of meta-analysis.**


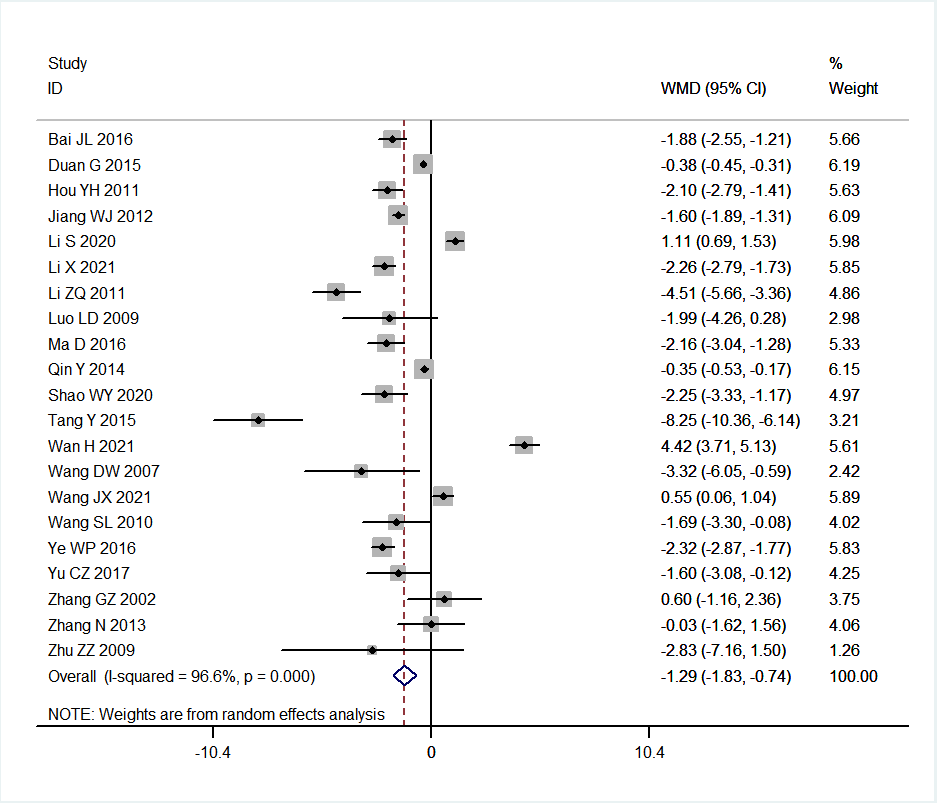


**S15 Fig. Funnel plot of *Fuling* formulae plus hypoglycaemic agents versus hypoglycaemic agents alone for fasting insulin levels**

**
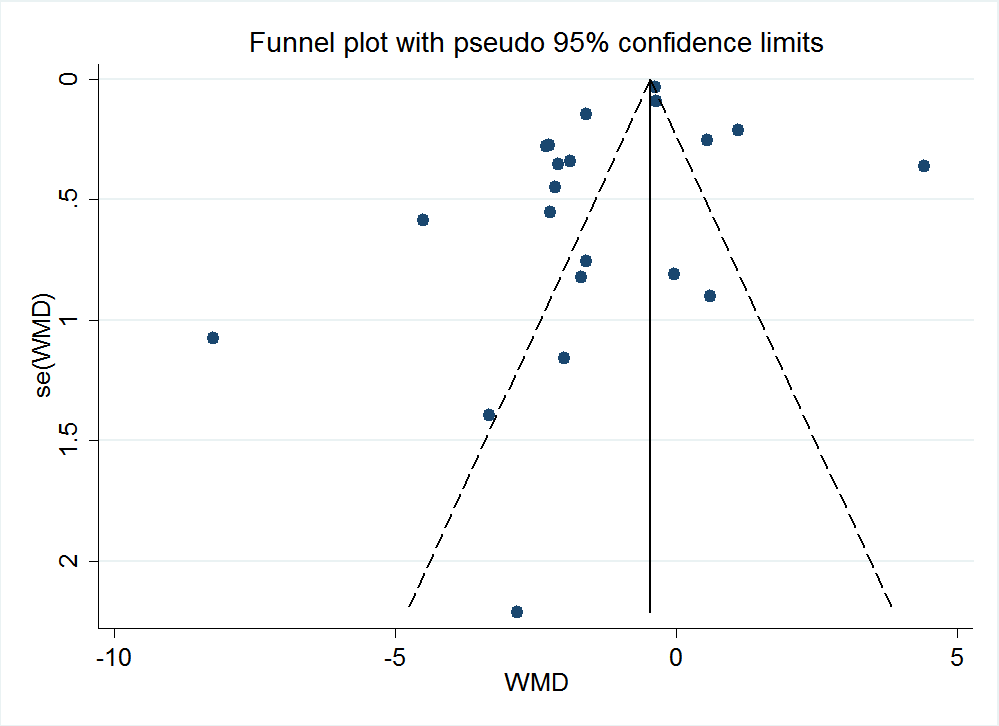
**

**S16 Fig. Forest plot of homeostatic model assessment of insulin resistance at end of treatment, *Fuling* formulae plus hypoglycaemic agents versus hypoglycaemic agents alone, results of meta-analysis.**

**
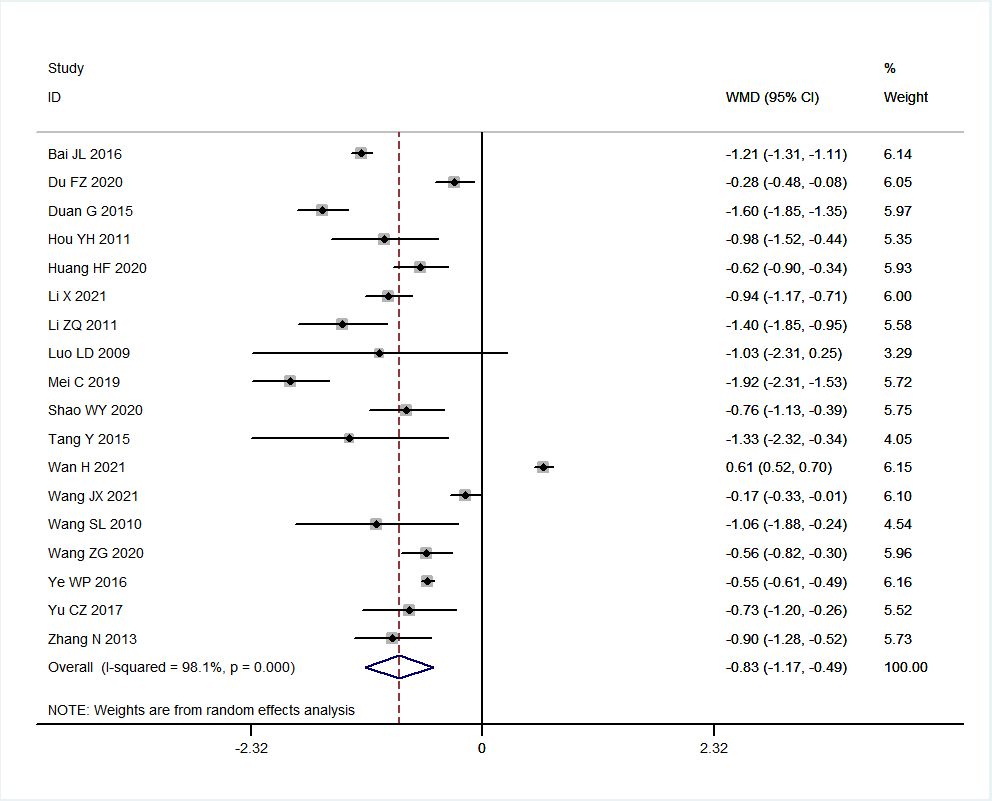
**

**S17 Fig. Funnel plot of *Fuling* formulae plus hypoglycaemic agents versus hypoglycaemic agents alone for the homeostatic model assessment of insulin resistance**

**
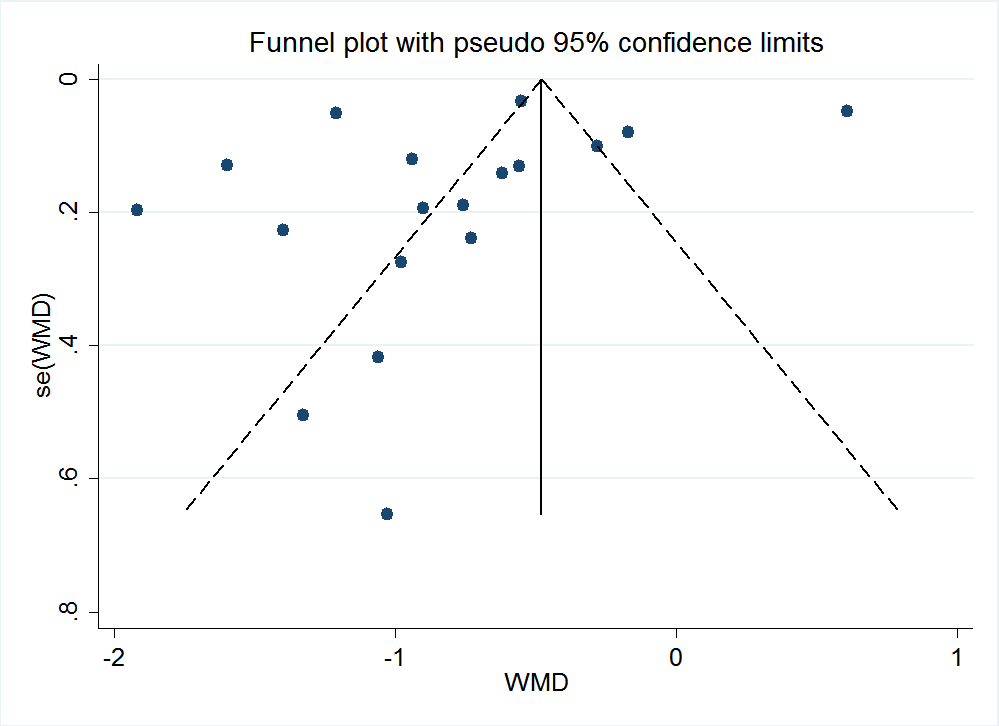
**

**S18 Fig. Forest plot of insulin resistance at end of treatment, *Fuling* formulae plus hypoglycaemic agents versus hypoglycaemic agents alone, results of meta-analysis.**

**
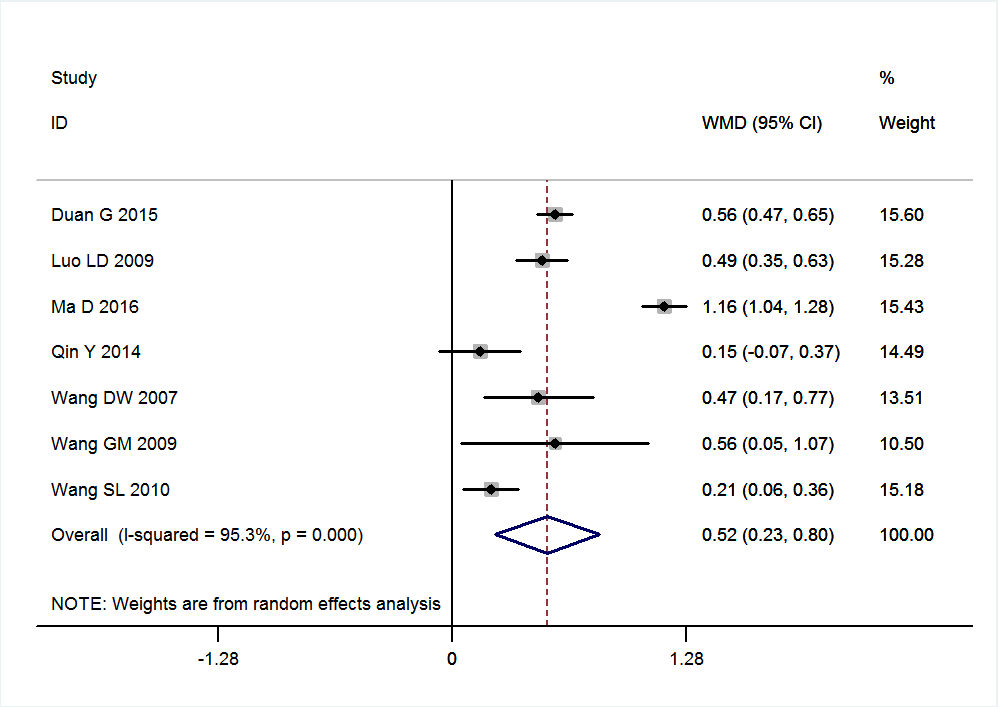
**

**S19 Fig. Forest plot of body mass index at end of treatment, *Fuling* formulae plus hypoglycaemic agents versus hypoglycaemic agents alone, results of meta-analysis.**

**
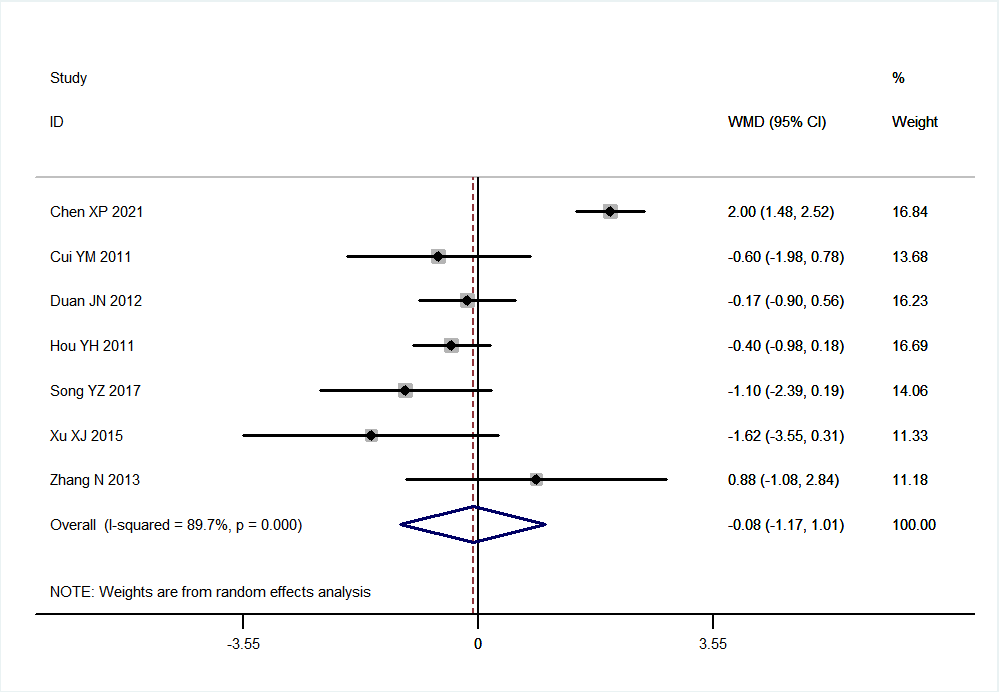
**

**S20 Fig. Forest plot of adverse events at end of treatment, *Fuling* formulae plus hypoglycaemic agents versus hypoglycaemic agents alone, results of meta-analysis.**

**
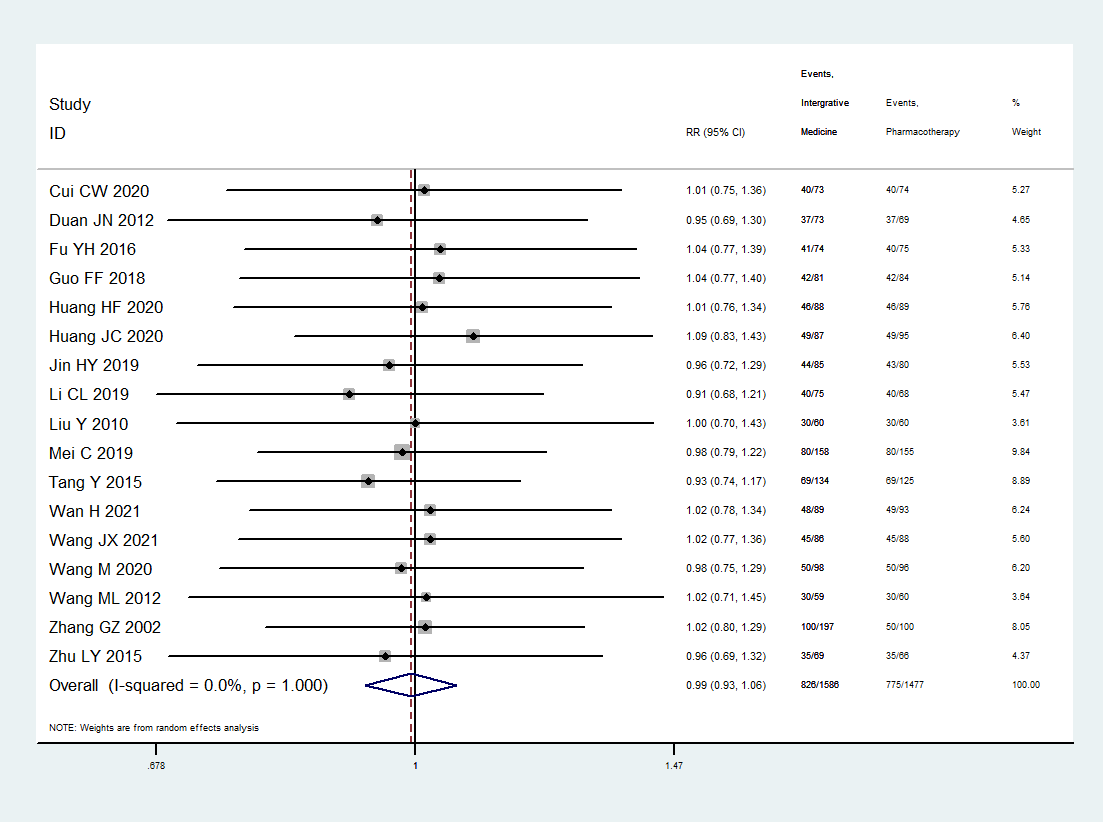
**
